# Novel pretreatment nomograms based on pan-immune-inflammation value for predicting clinical outcome in patients with head and neck squamous cell carcinoma

**DOI:** 10.3389/fonc.2024.1399047

**Published:** 2024-06-10

**Authors:** Qian Chen, Shi-Yang Wang, Yue Chen, Ming Yang, Kai Li, Zi-Yang Peng, Chong-Wen Xu, Xiao-Bao Yao, Hong-Hui Li, Qian Zhao, Yu-Dan Cao, Yan-Xia Bai, Xiang Li

**Affiliations:** ^1^ Department of Otorhinolaryngology Head and Neck Surgery, The First Affiliated Hospital of Xi’an Jiaotong University, Xi’an, Shaanxi, China; ^2^ Center for Gut Microbiome Research, Med-X Institute Centre, The First Affiliated Hospital of Xi’an Jiaotong University, Xi’an, Shaanxi, China; ^3^ School of Future Technology, National Local Joint Engineering Research Center for Precision Surgery and Regenerative Medicine, Xi’an Jiaotong University, Xi’an, Shaanxi, China

**Keywords:** head and neck squamous cell carcinoma, pan-immune-inflammation value, nomogram, disease-free survival, prognostic model

## Abstract

**Background:**

The prognostic value of an effective biomarker, pan-immune-inflammation value (PIV), for head and neck squamous cell carcinoma (HNSCC) patients after radical surgery or chemoradiotherapy has not been well explored. This study aimed to construct and validate nomograms based on PIV to predict survival outcomes of HNSCC patients.

**Methods:**

A total of 161 HNSCC patients who underwent radical surgery were enrolled retrospectively for development cohort. The cutoff of PIV was determined using the maximally selected rank statistics method. Multivariable Cox regression and least absolute shrinkage and selection operator (LASSO) regression analyses were performed to develop two nomograms (Model A and Model B) that predict disease-free survival (DFS). The concordance index, receiver operating characteristic curves, calibration curves, and decision curve analysis were used to evaluate the nomograms. A cohort composed of 50 patients who received radiotherapy or chemoradiotherapy (RT/CRT) alone was applied for generality testing of PIV and nomograms.

**Results:**

Patients with higher PIV (≥123.3) experienced a worse DFS (HR, 5.01; 95% CI, 3.25–7.72; *p*<0.0001) and overall survival (OS) (HR, 5.23; 95% CI, 3.34–8.18; *p*<0.0001) compared to patients with lower PIV (<123.3) in the development cohort. Predictors of Model A included age, TNM stage, neutrophil-to-lymphocyte ratio (NLR), and PIV, and that of Model B included TNM stage, lymphocyte-to-monocyte ratio (LMR), and PIV. In comparison with TNM stage alone, the two nomograms demonstrated good calibration and discrimination and showed satisfactory clinical utility in internal validation. The generality testing results showed that higher PIV was also associated with worse survival outcomes in the RT/CRT cohort and the possibility that the two nomograms may have a universal applicability for patients with different treatments.

**Conclusions:**

The nomograms based on PIV, a simple but useful indicator, can provide prognosis prediction of individual HNSCC patients after radical surgery and may be broadly applicated for patients after RT/CRT alone.

## Introduction

Head and neck squamous cell carcinoma (HNSCC) is the sixth most common cancer worldwide, which accounts for the largest proportion of head and neck malignancies (1). HNSCC, stemming from the epithelial cells in the mucosal epithelia of the oral cavity, nasopharynx, oropharynx, larynx, hypopharynx, and sinonasal tract, was globally reported to have 879,000 new cases and 445,000 deaths in 2020 (2–4). According to TNM stage and primary site, surgery, radiotherapy (RT), and chemotherapy (CT) or in combination are utilized as curative treatments (2). Generally, radical surgery alone or RT alone is applied for early stages, and postoperative radiotherapy (PORT) or postoperative chemoradiotherapy (POCRT) is applied for advanced stages. In addition, RT or CRT alone is also applied for patients who had lost the opportunity of surgery or given up surgery (2). Despite radical resection surgery, RT or concurrent chemoradiation (CRT) has been applied in many cases; approximately 40% of the patients will develop a locoregional recurrence or distant metastasis with a higher risk of mortality within 5 years, suggesting that prognostic stratification and prediction of treatment outcomes are essential (5–7).

The TNM staging system (the 8th edition) published by the American Joint Committee on Cancer (AJCC) is most regularly used to evaluate the prognosis of HNSCC patients (8, 9); however, its major limitations include neglecting other key factors (such as immune and inflammation status), relatively low accuracy, and poor performance in predicting individual survival and recurrence risk after curative treatments (10). Therefore, a personalized prediction model is needed for treated HNSCC patients.

Since immunity and inflammation have been confirmed as critical roles in initiation and progression of many cancers (11, 12), a variety of immune-inflammatory biomarkers (IIBs) obtained from peripheral blood have been investigated in cancer patients, including neutrophil-to-lymphocyte ratio (NLR) (13), platelet-to-lymphocyte ratio (PLR) (14), and lymphocyte-to-monocyte ratio (LMR) (15), which showed a predictive relevance with patients’ survival. However, the clinical practicality of such single biomarkers is confined by their low discriminative ability. Considering the complicated network of interactions among immunity, inflammation, and cancer prognosis, we intended to search for a composite biomarker incorporating divergent immune-inflammatory populations and reflecting the holistic immunity and inflammation status. Hu et al. reported that systemic immune-inflammation index (SII), an integrated indicator based on neutrophil, lymphocyte, and platelet, had stronger discrimination ability with 0.66 of the area under the curves (AUC) for predicting survival and recurrence of hepatocellular carcinoma patients than NLR and PLR (16). Chao et al. found that another integrated indicator, systemic inflammation response index (SIRI) based on neutrophil, monocyte, and lymphocyte, had a larger AUC than NLR, PLR, and MLR whether for 3-year (SIRI, 0.61; NLR, 0.53; PLR, 0.56; MLR, 0.59) or 5-year (SIRI, 0.61; NLR, 0.55; PLR, 0.56; MLR, 0.59) cervical cancer patients survival rate, and SIRI was identified as the only independent prognostic factor by multivariate analysis (17). Pan-immune-inflammation value (PIV), a recently developed, more complex immune-inflammation biomarker derived from neutrophil, platelet, monocyte, and lymphocyte counts, has been proposed as a more reliable predictor with robust prognostic value in patients with colorectal cancer (18), small cell lung cancer (19), and other malignancies (20–22). Nevertheless, to the best of our knowledge, there is still no study assessing the prognostic ability of PIV in HNSCC patients treated with surgery or chemoradiotherapy, respectively.

With the promise of an estimated numerical prognosis for every patient, the use of nomogram has been meteorically rising for cancer prognosis in recent years (23, 24). It fulfills the goal of constructing an integrated model tailored to the profile of an individual patient, produces a marked effect in promoting personalized healthcare, and is convenient for clinicians to use in prognosis prediction (25–27), which might also benefit HNSCC patients in clinical application.

In our study, we investigated the potential role of PIV in predicting the outcomes of HNSCC patients receiving radical resection surgery and established PIV-based nomograms to visualize prognostic factors and facilitate clinical decision-making. Furthermore, a cohort composed of patients treated with RT/CRT was utilized for generality test to verify the prognostic value of PIV and wide applicability of the constructed nomograms.

## Materials and methods

### Patient selection and study design

A total of 231 patients who were diagnosed with HNSCC in the Department of Otorhinolaryngology Head and Neck, First Affiliated Hospital of Xi’an Jiaotong University from January 2008 to January 2018, were enrolled retrospectively for development cohort to construct nomograms. The inclusion criteria were as follows: (1)patients who underwent radical tumor resection surgery and had been pathologically diagnosed as HNSCC; (2) HNSCC had to be primary; and (3) patients with complete clinicopathological and follow-up records. The exclusion criteria were as follows: (1) patients with other malignant diseases previously diagnosed; (2) patients with a history of inflammatory, autoimmune disease, and hematological disease; and (3) patients who had a postoperative survival time <1 month.

In addition, 50 HNSCC patients who only received radiotherapy or chemoradiotherapy after diagnosis between March 2008 and December 2017 in the same hospital were supplemented as a RT/CRT cohort to test the general utility of constructed nomograms based on PIV. Patients who underwent surgery died within 1 month after therapy were excluded. Other inclusion and exclusion criteria were the same with the development cohort.

All data collection was conducted in accordance with the Declaration of Helsinki and approved by the ethics committee of First Affiliated Hospital of Xi’an Jiaotong University (No. 2022–321). All participants signed an informed consent.

### Data collection

Smoking index was calculated as the average number of cigarettes smoked per day multiplied by the number of years smoking (28). The TNM stage was in compliance with the 8th AJCC TNM stage system (8). Blood samples were collected and analyzed within 7 days before surgery or the start date of RT/CRT. We defined normal level of fibrinogen (FIB), albumin (ALB), and total bilirubin levels (TBIL) as 2–4 g/L, 40–55 g/L, and 3.4–17.1 µmol/L, respectively. Immune-inflammatory indices were calculated as NLR = neutrophil count (10^9^/L)/lymphocyte count (10^9^/L) (13); PLR = platelet count (10^9^/L)/lymphocyte count (10^9^/L) (14); LMR = lymphocyte count (10^9^/L)/monocyte count (10^9^/L) (15); and PIV was calculated as previously described: [neutrophil count (10^9^/L) × platelet count (10^9^/L) × monocyte count (10^9^/L)]/lymphocyte count (10^9^/L) (18–22).

For the sake of collecting clinical outcomes information, periodical follow-up evaluations were conducted for all patients until death. After surgery or chemoradiotherapy, patients were rechecked for recurrence by physical examination, blood test, and fiberoptic pharyngorhinoscopy every 2 months in the first 2 years, every 6 months for 3–5 years, and thereafter once a year. CT scans of the neck and chest were performed every 6 months for 3 years and once a year thereafter. Hypopharyngeal cancer patients were additionally monitored by esophagogastroduodenoscopy once a year. Biopsy or fine needle aspiration cytology was carried out if there was suspicious of local or regional recurrences.

The primary endpoint for development cohort was disease-free survival (DFS), which was defined as the interval time (in months) between the radical surgery and the date of death or recurrence, or final follow-up (on 1 January 2023), whichever came first. Progression-free survival (PFS), the primary endpoint for the RT/CRT cohort, was measured in months from the start date of RT/CRT to the date of disease progression or death from any cause or the censoring date of last follow-up (on 30 September 2023). The secondary endpoint, overall survival (OS), was calculated as the time (in months) from the first definite diagnosis to the date of death or final follow-up, whichever came first.

### Statistical analysis

All data were statistically analyzed using IBM SPSS 26.0 and R software (version 4.2.2) with the assistance of R studio (version 2022.07.2 + 576). Restricted cubic spline (RCS) regression was performed to evaluate the association between DFS and PIV as a continuous variable. The cutoff point was determined using maximally selected rank statistics. The differences in the clinicopathological characteristics between the low and high PIV groups were tested by one-way analysis of variance (ANOVA; normal distribution), Wilcoxon rank sum test (skewed distribution), or Chi-square test (categorical variables). Spearman correlation analysis was used to test correlations.

We performed the Kaplan–Meier analysis and log-rank test to plot the survival curves. Subgroup analysis and interaction terms were used to confirm whether there were any correlations between PIV and the various clinical parameters. Univariate and multivariate Cox regression using stepwise backward LR method and the least absolute shrinkage and selection operator (LASSO) regression model were used to search for independent prognostic factors of convincing nomograms in HNSCC patients. The LASSO coefficient profiles of the 19 variables for development cohort were constructed from the log (λ) sequence, which shrank exactly to zero to be used for variable selection. A string of λ values covering the entire range, which were determined by 1,000-fold bootstrapping resampling, was elected to calculate the cross-validation error and search out prognostic factors of the nomogram. The concordance index (C-index) based on Harrell C statistics and receiver operating characteristic (ROC) curve with AUC were used to measure the accuracy of the nomograms. The relationship between the predicted and actual risks for outcomes of the nomograms was graphically displayed via calibration plots, while decision curve analysis (DCA) was used to evaluate the clinical benefits and utility of the nomogram for predicting prognosis. Trend tests were performed by modeling PIV as a continuous variable, dividing it by cutoff point or into quartiles; Wald tests were used to assess statistical significance. A *p*-value <0.05 from the two-sided test was considered statistically significant.

## Results

### Patient clinicopathological characteristics

A total of 231 HNSCC patients after radical surgery were screened for eligibility, 161 of whom [151 (93.8%) men and 10 (6.2%) women] were eventually enrolled in development cohort. In these patients, 91 (56.5%) were over 60 years at diagnosis and 105 (65.2%) had a smoking index of <650. The primary tumors of the vast majority were located in the larynx [137 (85.1%)] and nearly half [74 (46%)] were well differentiated. Additionally, the distribution of the TNM stage at diagnosis was 0/I, 43.5%; II, 14.9%; III, 17.4%; and IV, 24.2%, respectively. It is worth noting that, compared to those with stage 0/I, HNSCC patients with higher TNM stages harbored significantly higher PIV and log PIV (0/I vs. II, *p*<0.001; 0/I vs. III, *p*<0.01; 0/I vs. IV, *p*<0.001) (**Supplementary Figure 1**). Besides radical tumor resection, 56 (34.8%) patients received PORT or POCRT and others received radical surgery only. The proportion of patients with normal levels of FIB, ALB, and TBIL were 80.7%, 50.9%, and 86.3%, respectively. The median values of the NLR, PLR, and LMR for all patients were 1.94, 97.26, and 4.43, respectively. Details are given in **Table 1**.

**Table 1:** Baseline clinicopathological characteristics of the development cohort.

Characteristic	Total Patients	PIV-Low	PIV-High	*p*-value
N (%)	161	76 (47.2)	85 (52.8)	
Sex, N (%)				0.244
Male	151 (93.8)	69 (90.8)	82 (96.5)	
Female	10 (6.2)	7 (9.2)	3 (3.5)	
Age (year), N (%)				0.346
<60	70 (43.5)	36 (47.4)	34 (40.0)	
≥60	91 (56.5)	40 (52.6)	51 (60.0)	
Smoking index, N (%)				0.142
<650	105 (65.2)	54 (71.1)	51 (60.0)	
≥650	56 (34.8)	22 (28.9)	34 (40.0)	
Tumor type, N (%)				0.196
Laryngeal cancer	137(85.1)	61 (80.3)	76 (89.4)	
Hypopharyngeal cancer	19 (11.8)	11 (14.5)	8 (9.4)	
Other types	5 (3.1)	4 (5.3)	1 (1.2)	
Tumor differentiation, N (%)				0.286
Well differentiated	74 (46.0)	30 (39.5)	44 (51.8)	
Moderately differentiated	69 (42.9)	37 (48.7)	32 (37.6)	
Poorly differentiated	18 (11.2)	9 (11.8)	9 (10.6)	
T stage, N (%)				<0.001
Tis/T1	77 (47.8)	52 (68.4)	24 (28.2)	
T2	36 (22.4)	12 (15.8)	25 (29.4)	
T3	38 (23.6)	8 (10.5)	30 (35.3)	
T4	10 (6.2)	4 (5.3)	6 (7.1)	
N stage, N (%)				0.134
N0	113 (70.2)	57 (75.0)	56 (65.9)	
N1	14 (8.7)	8 (10.5)	6 (7.1)	
N2	34 (21.1)	11 (14.5)	23 (27.1)	
TNM stage (AJCC, 8th), N (%)				<0.001
0/I	70 (43.5)	47 (61.8)	23 (27.1)	
II	24 (14.9)	7 (9.2)	17 (20.0)	
III	28 (17.4)	10 (13.2)	18 (21.2)	
IV	39 (24.2)	12 (15.8)	27 (31.8)	
PORT/POCRT				0.033
Undone	105 (65.2)	56 (73.7)	49 (57.6)	
Done	56 (34.8)	20 (26.3)	36 (34.8)	
FIB, N (%)				0.146
Normal	130 (80.7)	65 (85.5)	65 (76.5)	
Abnormal	31 (19.3)	11 (14.5)	20 (23.5)	
ALB, N (%)				0.175
Normal	82 (50.9)	43 (56.6)	39 (45.9)	
Abnormal	79 (49.1)	33 (43.4)	46 (54.1)	
TBIL, N (%)				0.458
Normal	139 (86.3)	64 (84.2)	75 (88.2)	
Abnormal	22 (13.7)	12 (15.8)	12 (11.8)	
LYM (10^9^/L), median (IQR)	1.76 (1.39–2.15)	1.76 (1.44–2.32)	1.76 (1.30–2.10)	0.169 ^*^
MON (10^9^/L), median (IQR)	0.39 (0.30–0.53)	0.31 (0.26–0.39)	0.50 (0.39–0.59)	<0.001 ^*^
NEU (10^9^/L), median (IQR)	3.39 (2.83–4.36)	2.92 (2.48–3.36)	4.15 (3.42–5.32)	<0.001 ^*^
PLT (10^9^/L), mean ± SD	181.59 ± 54.52	161.08 ± 44.36	199.93 ± 56.42	<0.001 ^#^
NLR, median (IQR)	1.94 (1.42–2.74)	1.44 (1.22–2.00)	2.49 (1.90–3.34)	<0.001 ^*^
PLR, median (IQR)	97.26 (74.25–128.86)	84.68 (66.33–109.79)	114.84 (85.53–168.97)	<0.001 ^*^
LMR, median (IQR)	4.43 (3.18–6.00)	6.03 (4.66–7.23)	3.48 (2.54–4.40)	<0.001 ^*^

^*^Wilcoxon rank sum test, ^#^one-way analysis of variance, others are Chi-square test. Data are represented as mean (SD), median (interquartile range) or number (%).

PORT, postoperative radiotherapy; POCRT, postoperative chemoradiotherapy; FIB, fibrinogen; ALB, albumin; TBIL, total bilirubin; LYM, lymphocyte; MON, monocyte; NEU, neutrophil; PLT, platelet; NLR, neutrophil-to-lymphocyte ratio; PLR, platelet-to-lymphocyte ratio; LMR, lymphocyte-to-monocyte ratio; PIV, pan-immune-inflammation value; IQR, interquartile range; SD, standard deviation.

### The optimal cutoff value for PIV

When analyzed as a continuous variable, the restricted cubic spline plot showed that PIV had a positive dose–response association with the mortality and recurrence risk in development cohort, and the risk increased rapidly when PIV value exceeded 123.3, suggesting its potential to conjecture patients’ survival (**Supplementary Figure 2A**). Considering the continuity of PIV and its association with DFS, we performed a bilateral outcome-oriented maximally selected rank statistics test, indicating that the optimal cutoff value for PIV associated with DFS was 123.3 (**Supplementary Figure 2B**). All patients in the development cohort were then stratified into two groups based on the cutoff of PIV.

### Correlation of PIV with clinicopathological characteristics of HNSCC patients

Ultimately, 76 patients in PIV-Low group and 85 patients in PIV-High group were analyzed. As shown in **Table 1**, higher PIV was significantly associated with higher T stage, more advanced TNM stage, receiving PORT/POCRT, higher NLR, higher PLR, and lower LMR (*p*<0.001, *p*<0.001, *p*=0.033, *p*<0.001, *p*<0.001, and *p*<0.001, respectively), while no statistically relevant correlations were noted in other characteristics. In addition, Spearman rank correlation test revealed that FIB level had a strong positive association with PIV when grouped by age (<60 years: R=0.321, *p*=0.007; ≥60 years: R=0.466, *p*<0.001), however, other clinical parameters did not exhibit this feature (**Supplementary Figure 3**).

### Kaplan–Meier survival analysis

**Figure 1:**
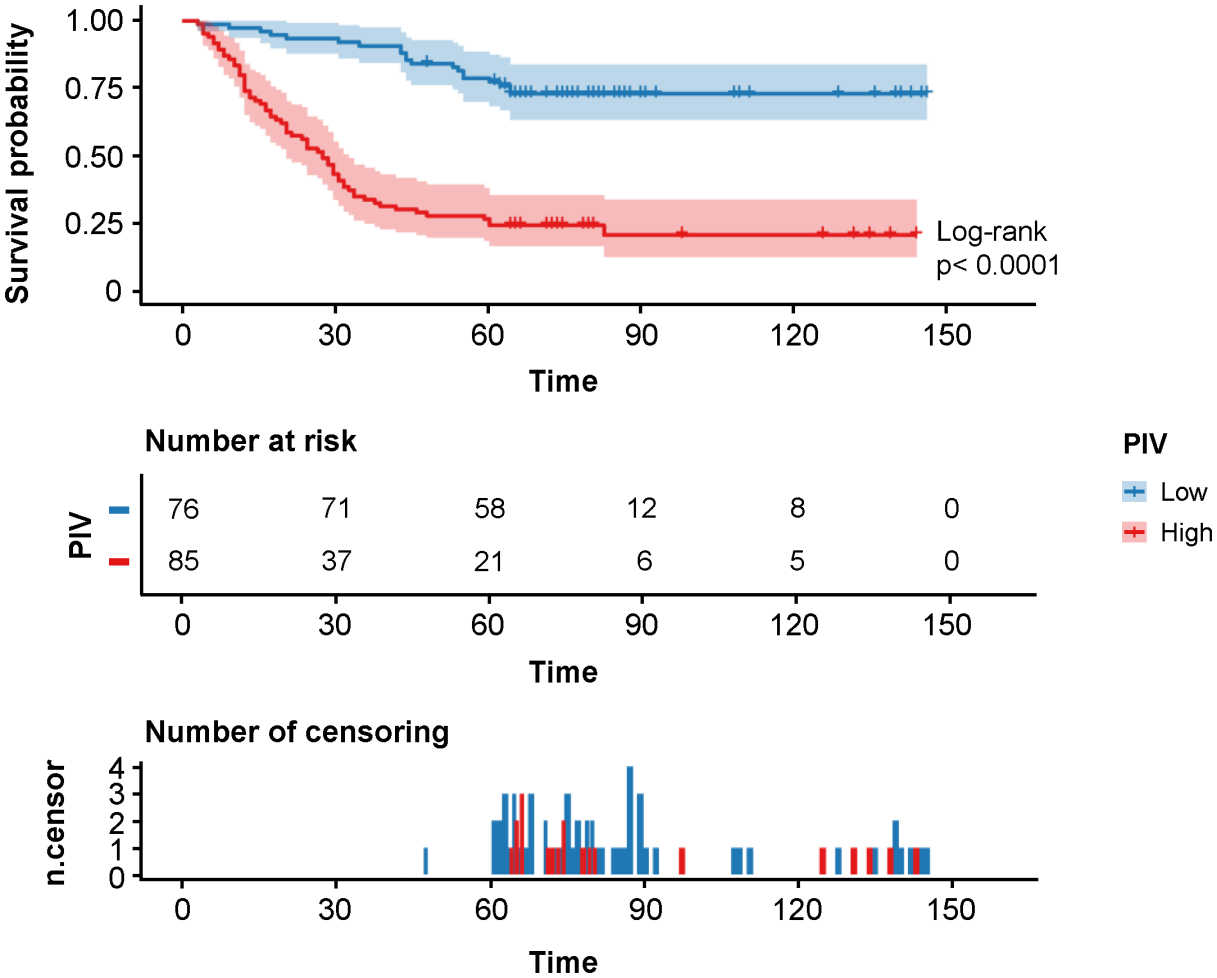
Kaplan–Meier survival analysis of DFS in the different PIV (Low and High) groups of the development cohort.

At data cutoff of the development cohort, the median follow-up period was 60 months (range, 3–143 months), during which 79 patients died and 18 patients experienced tumor recurrence, and the median DFS and OS were 59 months and 60 months, respectively. The DFS rates at 1 year, 3 years, and 5 years were 88.2%, 60.9%, and 49.1% as compared with 90.7%, 60.9%, and 50.3% of the OS rates, respectively. In terms of Kaplan–Meier curves and log-rank test results, the 5-year DFS rate of PIV-Low group was statistically higher than that of PIV-High group (73.7% vs. 23.5%, HR: 5.00, 95% CI: 3.23–7.71, *p*<0.0001, **Figure 1**), which was similar to that of the 5-year OS rate (76.3% vs. 27.1%, HR: 5.29, 95% CI: 3.38–8.28, *p*<0.0001, **Supplementary Figure 4**). All these results indicated that PIV-High group had a worse prognosis compared with PIV-Low group.

### Prognostic evaluation of PIV in subgroup analysis

To further investigate the survival characteristics in specific TNM stages, we divided the development cohort into four groups and found that higher PIV was still correlated with poorer disease-free survival in stage 0/I (93.6% vs. 34.8%, HR: 14.98, 95% CI: 5.16–43.53, *p*<0.0001) and III (50.0% vs. 16.7%, HR: 3.22, 95% CI: 1.34–7.73, *p*=0.013, **Supplementary Figures 5A, C**) patients. Despite that similar trends were observed in stage II and IV patients, the results did not reach statistical significance (stage II: 71.4% vs. 29.4%, HR: 3.59, 95% CI: 1.22–10.60, *p*=0.069; stage IV: 16.7% vs. 14.8%, HR: 1.86, 95% CI: 0.94–3.69, *p*=0.083, **Supplementary Figures 5B, D**). As shown in **Supplementary Figure 6**, the result was analogously applicable to OS in stage 0/I (95.7% vs. 43.5%, HR: 18.47, 95% CI: 5.83–58.54, *p*<0.0001), III (50.0% vs. 16.7%, HR: 3.22, 95% CI: 1.34–7.73, *p*=0.013), and IV (33.3% vs. 14.8%, HR: 2.20, 95% CI: 1.08–4.47, *p*=0.041) patients, while it was a pity that the result was not statistically significant in stage II patients (57.1% vs. 35.3%, HR: 2.49, 95% CI: 0.86–7.25, *p*=0.140).

Further stratified analyses were conducted, disclosing the associations between PIV and HR for DFS and OS in various subgroups according to sex, age, smoking index, T stage, N stage, TNM stage, PORT/POCRT, FIB, ALB, and TBIL (**Supplementary Tables 1**, **2**). Interaction analyses indicated that N stage (*p*=0.004), TNM stage (*p*=0.035), and PORT/POCRT (*p*=0.006) were able to interact with PIV for DFS, while no interactions between PIV and other clinicopathological characteristics were observed (**Supplementary Table 1**). Specifically, stage N1, stage 0/I, and receiving no PORT/POCRT were associated with higher risk for death and recurrence. The significant interactions in subgroups defined by N stage (*p*=0.031) and PORT/POCRT (*p*=0.024) were also detected for OS (**Supplementary Table 2**).

### Cox regression analyses and LASSO regression analysis

Univariate and multivariate Cox regression analyses were first performed to investigate potential prognostic factors for DFS and OS in development cohort (**Table 2**; **Supplementary Table 3**). According to univariate analysis, the following variables were proved statistically affecting both DFS and OS: age, smoking index, T stage, N stage, TNM stage, PORT/POCRT, FIB, ALB, NLR, PLR, LMR, and PIV. The multivariate analysis that included all factors whose *p <*0.1 in the univariate analysis indicated that higher age (DFS: *p*=0.039; OS: *p*=0.010), higher TNM stage (DFS: *p*<0.001; OS: *p*<0.001), higher NLR (DFS: *p*=0.009; OS: *p*=0.007), and higher PIV (DFS: *p*=0.002; OS: *p*=0.005) were identified as independent prognostic factors for DFS and OS. Stage III and IV were stronger prognostic factors than PIV. Except for this, the HR of higher PIV for DFS (HR: 3.605, *p*<0.001) and OS (HR: 3.600, *p*<0.001) increased markedly, conforming that PIV was an important prognostic factor for DFS and OS except for TNM stage.

**Table 2:** Univariate and multivariate analyses of DFS according to clinicopathological factors in the development cohort.

Characteristic	Univariate analysis		Multivariate analysis	
	HR (95% CI)	*p*-value	HR (95% CI)	*p*-value
Sex				
Female	Ref			
Male	1.012 (0.410–2.499)	0.979		
Age (year)				
<60	Ref			
≥60	1.876 (1.192–2.952)	0.007	1.790 (1.119–2.863)	0.015
Smoking index				
<650	Ref			
≥650	1.616 (1.050–2.486)	0.029		
Tumor type				
Laryngeal cancer	Ref	0.571		
Hypopharyngeal cancer	0.784 (0.392–1.566)	0.490		
Other types	0.548 (0.134–2.232)	0.401		
Tumor differentiation				
Well differentiated	Ref	0.320		
Moderately differentiated	1.020 (0.643–1.620)	0.932		
Poorly differentiated	1.593 (0.845–3.002)	0.150		
T stage				
Tis/T1	Ref	<0.001		
T2	3.523 (1.993–6.227)	<0.001		
T3	3.744 (2.150–6.521)	<0.001		
T4	5.662 (2.606–12.305)	<0.001		
N stage				
N0	Ref	<0.001		
N1	2.343 (1.210–4.535)	0.012		
N2	2.795 (1.742–4.485)	<0.001		
TNM stage (AJCC, 8th)				
0/I	Ref	<0.001	Ref	<0.001
II	3.510 (1.743–7.069)	<0.001	2.308 (1.108–4.809)	0.025
III	4.198 (2.212–7.966)	<0.001	3.021 (1.574–5.798)	0.001
IV	5.838 (3.271–10.421)	<0.001	3.614 (1.992–6.554)	<0.001
PORT or POCRT				
Undone	Ref			
Done	2.499 (1.629–3.834)	<0.001		
FIB				
Normal	Ref			
Abnormal	2.144 (1.324–3.471)	0.002		
ALB				
Normal	Ref			
Abnormal	1.627 (1.057–2.504)	0.027		
TBIL				
Normal	Ref			
Abnormal	0.895 (0.475–1.687)	0.732		
NLR	1.248 (1.165–1.338)	<0.001	1.156 (1.058–1.262)	0.001
PLR	1.008 (1.005–1.012)	<0.001		
LMR	0.673 (0.583–0.775)	<0.001		
PIV				
Low (<123.3)	Ref			
High (≥123.3)	5.278 (3.148–8.848)	<0.001	3.605 (2.109–6.162)	<0.001

Ref, reference.

**Figure 2:**
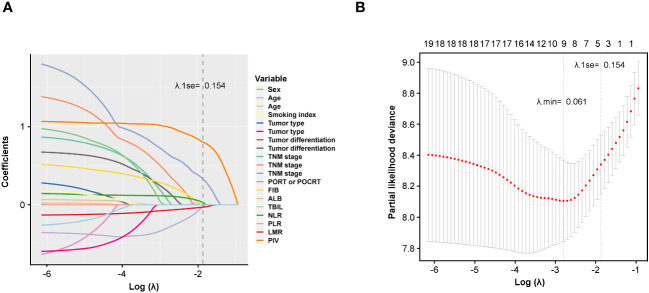
Potential prognostic factors selection using the LASSO regression model. **(A)** LASSO coefficient profiles of the 19 variables constructed from the log (λ) sequence; **(B)** 1,000-fold bootstrapping resampling cross-validation for tuning variable selection in the LASSO model. The number of variables was filtered by drawing dotted vertical lines at λ.min (left dotted line) and λ.1se (right dotted line), respectively, according to the minimum criterion. SE, standard error; min, minimum.

Furthermore, LASSO regression model was also applied to identify potential prognostic factors for DFS of the development cohort (**Figure 2**). Based on the purpose of constructing a handy prognosis model, the λ that provided the most parsimonious model within one standard error of the optimum value (λ.1se) was slated (**Figure 2A**). Ultimately, the cross-validation screened out three indicators based on λ.1se (0.154) including TNM stage, LMR, and PIV, which could be used for prognosis prediction (**Figure 2B**).

### Development and internal validation of nomograms

**Figure 3:**
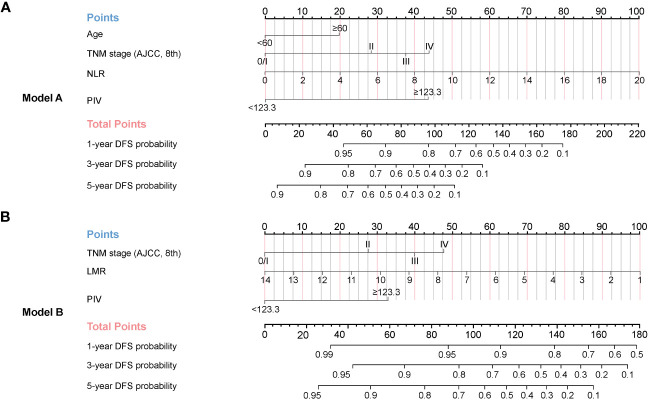
Nomograms established for 1-year, 3-year, and 5-year DFS in the development cohort. **(A)** Model A (based on multivariate Cox regression model) was adjusted for age, TNM stage, NLR, and PIV. **(B)** Model B (based on LASSO regression model) was adjusted for TNM stage, LMR, and PIV.

In order to precisely predict 1-year, 3-year, and 5-year DFS for HNSCC patients after radical resection, Model A and Model B were developed based on the results of the multivariate Cox regression analysis (**Figure 3A**) and LASSO regression model (**Figure 3B**), respectively. With each variable scored using the developed model, the 1-year, 3-year, and 5-year probability of DFS for each patient could be estimated by calculating the total score and drawing a vertical line to the probability scale axes.

**Figure 4:**
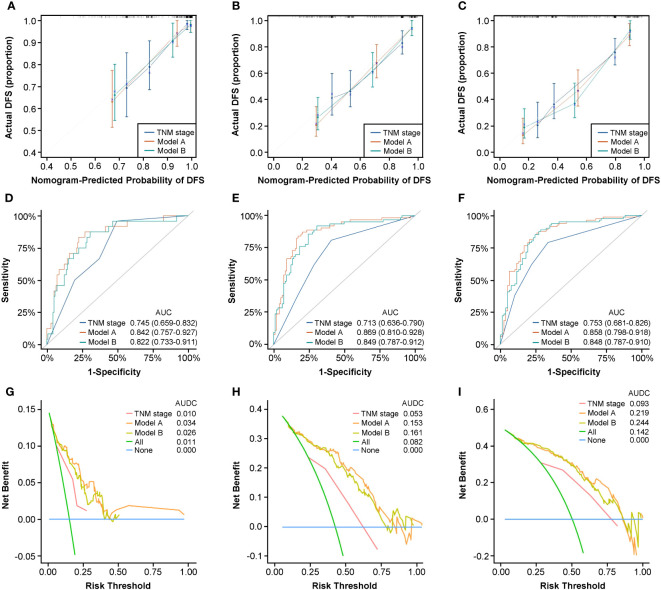
Nomograms internal validation according to bootstrapping method for 1-year, 3-year, and 5-year DFS in the development cohort. Calibration curves for 1-year **(A)**, 3-year **(B)**, and 5-year **(C)** DFS prediction based on TNM stage, Model A, and Model **(B)** ROC analyses of 1-year **(D)**, 3-year **(E)**, and 5-year **(F)** DFS prediction based on TNM stage, Model A, and Model **(B)** DCA curves for 1-year **(G)**, 3-year **(H)**, and 5-year **(I)** DFS prediction based on TNM stage, Model A, and Model **(B)** ROC, receiver operating characteristic curve; DCA, decision curve analysis.

Several criteria regarding discrimination and calibration were used to thoroughly evaluate and validate prediction performance of Model A and Model B internally. The C-indexes of the two models both performed better than that of traditional AJCC TNM stage in internal validation (Model A, 0.795; Model B, 0.788; TNM stage, 0.691). The calibration curves for the two models showed satisfactory consistency between actual observations and nomogram predictions for 1-year, 3-year, and 5-year DFS (**Figures 4A–C**) and OS (**Supplementary Figures 7A–C**) in internal validation. In addition, the ROC curves for Model A and Model B in internal validation showed superior sensitivity and specificity for DFS (**Figures 4D–F**) and OS (**Supplementary Figures 7D–F**), reflecting good discrimination ability compared to AJCC TNM staging alone. Moreover, according to the results of DCA curves in internal validation, the clinical net benefits for DFS (**Figures 4G–I**) and OS (**Supplementary Figures 7G–I**) of Model A and Model B were outstanding compared with AJCC TNM staging, demonstrating the feasibility of making more valuable judgments in clinical application. To sum up, all results internally validated the excellent predictive ability and accuracy of Model A and Model B in comparison with traditional AJCC TNM stage system.

### Sensitivity analysis

To confirm the prognostic potential of PIV for HNSCC patients after radical resection, we performed sensitivity analysis (**Supplementary Tables 4**, **5**) and found that PIV levels were positively associated with the death and recurrence of the development cohort in both Model A and Model B. First, grouped by the cutoff point (<123.3 vs. ≥123.3), the sterling prognostic probability of PIV was confirmed on the basis of HRs for DFS and OS obtained in Model A and Model B. Additionally, whether as continuous or divided into quartiles by PIV level, the results indicated that patients with a higher PIV had significantly worse DFS and OS than that of patients with a lower PIV.

### Generality testing of prognostic factors and nomograms

Considering that some HNSCC patients received RT or CRT only because of early stages or other conditions of losing the opportunity for surgery in clinical practice, we supposed that the independent prognostic factors for HNSCC patients after surgery still correlated with prognosis of patients after RT/CRT. For this purpose, we enrolled a RT/CRT cohort composed of 50 HNSCC patients who only received radiotherapy or chemoradiotherapy and applied it for testing the generality of the independent prognostic factors identified in the development cohort. The baseline clinicopathological characteristics of the RT/CRT cohort are listed in **Supplementary Table 6**. The primary and secondary endpoints of this cohort were PFS and OS, respectively.

For a start, we divided the cohort into PIV-Low and High groups by predetermined cutoff value and performed Kaplan–Meier analysis and log-rank test to identify the difference between the two groups. The results shown in **Supplementary Figure 8** confirm that PIV-High group did have worse PFS (2.8% vs. 78.6%, HR: 10.41, 95% CI: 5.51–19.69, *p*<0.0001) and OS (11.1% vs. 78.6%, HR: 9.82, 95% CI: 5.06–19.05, *p*<0.0001) than PIV-Low group.

Furthermore, we carried out univariate and multivariate Cox regression analyses to gain the prognostic factors for PFS and OS of the RT/CRT cohort. As shown in **Supplementary Table 7**, the result of multivariate Cox regression analysis verified that higher TNM stage (0/I: Ref, *p*=0.017; II: HR: 6.219, *p*=0.018; III: HR: 9.738, *p*=0.006; IV: HR: 13.945, *p*=0.001), higher PLR (HR: 1.007, *p*=0.002), and higher PIV (HR: 4.890, *p*=0.025) were independent prognostic factors for PFS of the RT/CRT cohort. As for OS, the result showed that higher smoking index (HR: 2.387, *p*=0.023), higher TNM stage (0/I: Ref, *p*=0.007; II: HR: 5.203 *p*=0.039; III: HR: 19.505, *p*=0.001; IV: HR: 14.773, *p*=0.001), higher PLR (HR: 1.008, *p*=0.003), and higher PIV (HR: 5.581, *p*=0.025) were independent prognostic factors (**Supplementary Table 8**).

According to the above results, higher TNM stage and higher PIV were important prognostic factors for both patients after radical surgery and patients after RT/CRT. Given that the nomograms, Model A and Model B, which both includes TNM stage and PIV, performed well in internal validation in the development cohort, we wanted to test if the nomograms have the wide application ability of prognosis prediction for patients who only received RT/CRT, in addition to patients received radical surgery.

**Figure 5:**
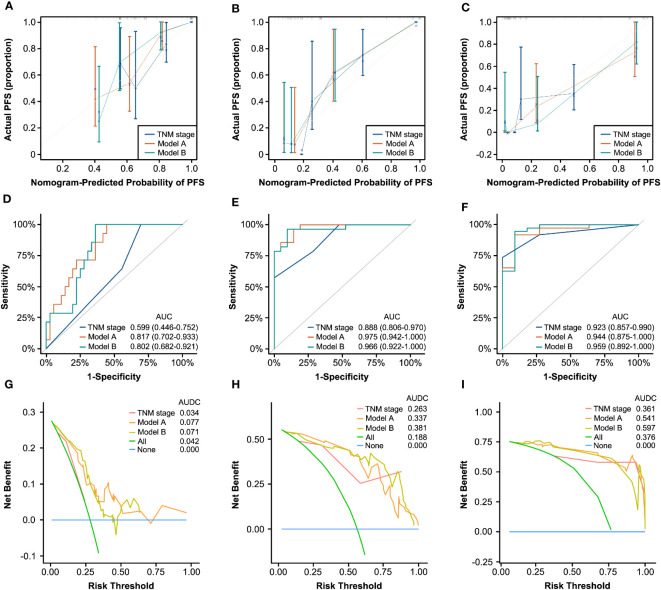
Generality testing of nomograms for 1-year, 3-year, and 5-year PFS in the RT/CRT cohort. Calibration curves for 1-year **(A)**, 3-year **(B)**, and 5-year **(C)** PFS prediction based on TNM stage, Model A, and Model **(B)** ROC analyses of 1-year **(D)**, 3-year **(E)**, and 5-year **(F)** PFS prediction based on TNM stage, Model A, and Model **(B)** DCA curves for 1-year **(G)**, 3-year **(H)**, and 5-year **(I)** PFS prediction based on TNM stage, Model A, and Model B.

We applied Model A and Model B for PFS and OS prediction in the RT/CRT cohort and evaluated the performance comprehensively. The C-indexes of the two models were both higher than that of traditional AJCC TNM stage (Model A, 0.815; Model B, 0.793; TNM stage, 0.683). The calibration curves for the two models showed satisfactory consistency between actual observations and nomogram predictions for 1-year, 3-year, and 5-year PFS (**Figures 5A–C**) and OS (**Supplementary Figures 9A–C**). In ROC analysis, the AUC values of Model A and Model B for PFS (**Figures 5D–F**) and OS (**Supplementary Figures 9D–F**) were obviously higher than that of AJCC TNM stage alone. In addition, the results of DCA curves showed that Model A and Model B could gain more clinical net benefits for PFS (**Figures 5G–I**) and OS (**Supplementary Figures 9G–I**) than AJCC TNM stage.

Based on these results, Model A and Model B showed good prediction performance for patients who only received RT/CRT compared with traditional AJCC TNM stage system. It indicated that in clinic, the two nomograms may have the ability of widespread clinical utility for HNSCC patients receiving different treatments including radical surgery or only RT/CRT.

## Discussion

In this study, we presented that a novel immune-inflammation blood-based composite biomarker, PIV, played a robust and independent prognostic role in patients with HNSCC receiving either radical surgery or RT/CRT alone. First, our results showed that higher PIV before surgery demonstrated an extensive and powerful association with poorer DFS and OS. Second, we found that PIV, partly associated with other clinicopathological parameters, had a positive dose–response relationship with mortality and recurrence risk among surgical patients. Furthermore, two nomograms separately based on multivariate Cox and LASSO regression models were constructed to predict the prognosis of patients with HNSCC. During the internal validation process, these two nomograms were thoroughly evaluated for prediction performance and showed bold discrimination and calibration capabilities. Of note, PIV had a relatively higher influence on survival in the regression models than the other canonical IIBs (i.e., NLR, PLR, and LMR) and was the only one variable that retained an independent prognostic role for DFS except for TNM staging system in the both two nomograms. What is more, the generality testing results showed that higher PIV were still related to worse PFS and OS, and the two nomograms embodied a possibility of wider application for patients treated with RT/CRT alone.

Recently, PIV, a systemic immune-inflammation score including four blood cell types (neutrophils, lymphocytes, platelets, and monocytes), was considered to have better prognostic value than other one-, two-, or three-component indexes (e.g., NLR, PLR, LMR, etc.) in patients with various tumor types, such as colorectal, renal, and breast cancer (18, 20, 29). The different components of PIV may reflect systemic immune activation and capture the different aspects of antitumor immunity, which explained the reason why PIV was superior to other IIBs. Up to now, only three studies have investigated the relationship of PIV with HNSCC. Yeh et al. presented that the cutoff for preoperative PIV of oral cavity squamous cell carcinoma (OSCC) was 268 and PIV was an independent prognostic factor for OS (HR: 1.281, *p*=0.027) and distant metastasis-free survival (HR: 1.274, *p*=0.031) (30). Guven et al. found that high PIV (>404) was associated with shorter OS (HR: 2.862, *p*=0.001) and DFS (HR: 2.485, *p*=0.002) in locally advanced HNSCC patients treated with chemoradiotherapy (31). Yilmaz et al. reported that the incidence of mandibular osteoradionecrosis (ORN) in the cohort with PIV≥ 833 is significantly higher than that in the cohort with PIV<833 (29.8% vs. 2.6%; *p*<0.001) of patients receiving chemoradiotherapy for locally advanced nasopharyngeal cancer (LA-NPC) (32). Our finding that PIV could serve as a valuable prognostic biomarker for predicting outcomes of HNSCC patients was in line with these previous studies. However, differed from these studies, our study included different subtypes of HNSCC patients and found an optimal cutoff for PIV (123.3) with good generality and accuracy for predicting various survival outcomes for patients undergoing either surgery (DFS: HR: 2.703, *p*=0.002; OS: HR: 2.586, *p*=0.005) or chemoradiotherapy (PFS: HR: 4.890, p=0.025; OS: HR: 5.581, *p*=0.025). It is no surprise that PIV cutoff values varied among different studies by reasons of differences in the study population and outcomes. Further subgroup analysis in our study confirmed the prognosis capacity of PIV for death and recurrence in subgroups of N stage, TNM stage, and PORT/POCRT. More importantly, two nomograms can be used as a good evaluation model for HNSCC patients who undergo radical surgery and may have the ability of predicting survival for patients treated with RT/CRT when surgery is not recommended in real-life clinical practice.

When applied clinically, the prognostic capacity of mortality and recurrence assessment as shown for each variable alone is limited due to the complexity and inhomogeneity of the cancer (33). Taking demographic and clinicopathological characteristics into consideration, the nomogram that integrates various key factors into a quantitative model has been certified to improve the prediction value and facilitate clinical application, outperforming other traditional evaluation indicators (33). Until today, there have been several researchers who have developed various nomograms based on immunological or inflammatory serological markers for evaluating prognosis of HNSCC patients. In a retrospective study including 169 patients with OSCC surgery, they created a nomogram consisting of TNM stage, age, LMR, and immunoglobulin G for predicting OS, with higher C-index than TNM stage (0.784 vs. 0.685) in internal validation (34). Another study by Peng Yeh et al. recruited 128 advanced oropharyngeal cancer (OPC) patients treated with chemoradiation and developed a nomogram combined with hemoglobin (Hb), SII, and SIRI for predicting disease-specific survival (DSS) (35). The predictive ability of the nomogram was only assessed by C-index (0.692) in internal validation. Compared to these two studies, the nomograms in our study exhibited several advantages. First, we constructed two nomograms based on multivariate Cox regression and LASSO regression analyses, respectively, for HNSCC patients undergoing radical surgery and made an internal appraisement and validation. Based on sensitivity analysis, different manifestations of PIV (as continuous, divided by cutoff, or by interquartile) played a crucial role in prognosis prediction for HNSCC patients in the two nomograms. In addition, we supplemented a RT/CRT cohort for generality testing of the two nomograms.

Conventionally, the AJCC tumor staging system is the top priority for predicting prognosis of HNSCC patients, whose stages of this system are generally considered to be strongly related with OS (36). Nevertheless, a considerable number of patients with the same stage were observed to present distinct prognoses, which may be explained by the fact that age, immunity, and inflammation status along with other factors are not included (10). Our nomograms could make up this deficiency appropriately to some extent. The composite biomarkers, SII and SIRI in the study of Peng Yeh et al. (35) and PIV in our study, performed better than LMR, PLR, and NLR in reflecting host immune and inflammatory status against cancer, which showed the feasibility and necessity to apply composite serological biomarkers in clinical practice. However, the underlying mechanism has not been comprehensively investigated, which needs further relevant studies to validate.

To our knowledge, this is the first study evaluating PIV’s predicting prognosis ability, constructing nomograms based on multivariate Cox regression and LASSO regression analyses for HNSCC patients after radical resection surgery and examining their general application ability for patients receiving RT/CRT alone, but it still has some limitations. First of all, owing to the retrospective nature of the study, additional prospective research is needed to confirm the prognosis value of PIV. Second, although internal validation of the nomograms showed satisfactory capability of discrimination and calibration, this study is a single-center study conducted in Chinese HNSCC patients with a relatively small sample size and still lacks external validation for reliability and practicality. Therefore, the generalization ability of the nomograms still requires more external validation using data from other research centers in different ethnic groups to eliminate the discrepancies in epidemiology and clinical behaviors among groups in different regions.

## Conclusions

In conclusion, our work identifies PIV as an inexpensive, minimally invasive prognostic biomarker that not is only associated with DFS and OS of HNSCC patients after radical surgery but also with PFS and OS of whom received RT/CRT alone. We constructed two nomograms incorporating PIV and key clinical features, which were internally validated as effective tools for individualized prediction of clinical outcomes for HNSCC patients.

## Data availability statement

The raw data supporting the conclusions of this article will be made available by the authors, without undue reservation.

## Ethics statement

The studies involving humans were approved by the ethics committee of First Affiliated Hospital of Xi’an Jiaotong University. The studies were conducted in accordance with the local legislation and institutional requirements. The participants provided their written informed consent to participate in this study.

## Author contributions

QC: Conceptualization, Methodology, Writing – original draft. S-YW: Methodology, Writing – original draft. YC: Data curation, Visualization, Writing – original draft. MY: Software, Writing – original draft. KL: Software, Writing – original draft. Z-YP: Validation, Writing – original draft. C-WX: Validation, Writing – original draft. X-BY: Formal Analysis, Writing –original draft. H-HL: Investigation, Writing – original draft. QZ: Resources, Writing – original draft. Y-DC: Data curation, Writing – original draft. Y-XB: Project administration, Supervision, Writing – review & editing. XL: Conceptualization, Funding acquisition, Project administration, Supervision, Writing –review & editing.

## Supplementary material

Supplementary Figure 1 Relationships between PIV and TNM stages in the development cohort. **(A)** The relationship between PIV and different TNM stages. **(B)** The relationship between Log PIV (natural-log transformed) and different TNM stages. **p<0.01; ***p<0.001. PIV, Pan-Immune-Inflammation value.

Supplementary Figure 2 Association of PIV with DFS in the development cohort. **(A)** The association between PIV (continuous) and hazard ratio of DFS based on restricted cubic spline plot. **(B)** The cut-off for PIV associated with DFS based on maximally selected rank statistics. DFS, disease-free survival; HR, hazard ratio.

Supplementary Figure 3 Relationships between PIV and clinical parameters in the development cohort. The clinical parameters include smoking index **(A)**, FIB **(B)**, ALB **(C)** and TBIL **(D)**, respectively. FIB, fibrinogen; ALB, albumin; TBIL, total bilirubin.

Supplementary Figure 4 Kaplan-Meier survival analysis of OS in the different PIV (Low and High) groups of the development cohort. OS, overall survival.

Supplementary Figure 5 Subgroup Kaplan-Meier survival analyses of DFS of the development cohort in the different PIV (Low and High) groups. All patients were stratified into four subgroups including TNM stage 0/I **(A)**, stage II **(B)**, stage III **(C)** and stage IV **(D)**.

Supplementary Figure 6 Subgroup Kaplan-Meier survival analyses of OS of the development cohort in the different PIV (Low and High) groups. All patients were stratified into four subgroups including TNM stage 0/I **(A)**, stage II **(B)**, stage III **(C)** and stage IV **(D)**.

Supplementary Figure 7 Nomograms internal validation according to bootstrapping method for 1, 3, 5-year OS in the development cohort. Calibration curves for 1-year **(A)**, 3-year **(B)** and 5-year (C) OS prediction based on TNM stage, Model A and Model B. ROC analyses of 1-year **(D)**, 3-year **(E)** and 5-year **(F)** OS prediction based on TNM stage, Model A and Model B. DCA curves for 1-year **(G)**, 3-year **(H)** and 5-year **(I)** OS prediction based on TNM stage, Model A and Model B.

Supplementary Figure 8 Kaplan-Meier survival analyses of DFS **(A)** and OS **(B)** in the different PIV (Low and High) groups of the RT/CRT cohort.

Supplementary Figure 9 Generality testing of nomograms for 1, 3, 5-year OS in the RT/CRT cohort. Calibration curves for 1-year **(A)**, 3-year **(B)** and 5-year **(C)** OS prediction based on TNM stage, Model A and Model B. ROC analyses of 1-year **(D)**, 3-year **(E)** and 5-year **(F)** OS prediction based on TNM stage, Model A and Model B. DCA curves for 1-year **(G)**, 3-year **(H)** and 5-year **(I)** OS prediction based on TNM stage, Model A and Model B.
