# Supplementary material for: Novel pretreatment nomograms based on pan-immune-inflammation value for predicting clinical outcome in patients with head and neck squamous cell carcinoma
Source: Front Oncol. 2024 Jun 10;14:1399047. doi: 10.3389/fonc.2024.1399047 (PMC11194608; doi:10.3389/fonc.2024.1399047)
Supplement: Supplementary file 1 [file Table_1.docx]

**Supplementary Table 1**

Associations between PIV (stratified by cut-off 123.3) and HR for DFS in various subgroups of the development cohort.

| **Characteristic** | **HR (95% CI)** | ***p*-value** | ***p* for interaction** |
| --- | --- | --- | --- |
| Sex |  |  | 0.695 |
| Female | 5.107 (0.820-31.788) | 0.080 |  |
| Male | 5.337 (3.138-9.077) | <0.001 |  |
| Age (year) |  |  | 0.193 |
| <60 | 3.434 (1.508-7.820) | 0.003 |  |
| ≥60 | 7.620 (3.954-14.687) | <0.001 |  |
| Smoking index |  |  | 0.196 |
| <650 | 6.641 (3.368-13.095) | <0.001 |  |
| ≥650 | 3.379 (1.575-7.249) | 0.002 |  |
| T stage |  |  | 0.499 |
| Tis/T1 | 7.202 (2.925-17.732) | <0.001 |  |
| T2 | 3.809 (1.402-10.351) | 0.009 |  |
| T3 | 3.280 (1.123-9.577) | 0.030 |  |
| T4 | 1.876 (0.478-7.367) | 0.367 |  |
| N stage |  |  | 0.004 |
| N0 | 11.461 (4.829-27.201) | <0.001 |  |
| N1 | 15.779 (1.809-137.636) | 0.013 |  |
| N2 | 1.734 (0.773-3.890) | 0.182 |  |
| TNM stage (AJCC, 8th) |  |  | 0.035 |
| 0/I | 16.310 (4.639-57.341) | <0.001 |  |
| II | 3.712 (0.821-16.779) | 0.088 |  |
| III | 3.489 (1.238-9.835) | 0.018 |  |
| IV | 1.926 (0.901-4.116) | 0.091 |  |
| PORT/POCRT |  |  | 0.006 |
| Undone | 9.633 (4.252-21.821) | <0.001 |  |
| Done | 2.475 (1.272-4.815) | 0.008 |  |
| FIB |  |  | 0.788 |
| Normal | 5.426 (3.015-9.763) | <0.001 |  |
| Abnormal | 4.345 (1.576-11.982) | 0.005 |  |
| ALB |  |  | 0.278 |
| Normal | 6.811 (3.078-15.069) | <0.001 |  |
| Abnormal | 4.028 (2.082-7.793) | <0.001 |  |
| TBIL |  |  | 0.832 |
| Normal | 5.122 (2.960-8.863) | <0.001 |  |
| Abnormal | 6.067 (1.582-23.263) | 0.009 |  |

Abbreviations: DFS, disease-free survival; HR, hazard ratio; CI, confidence interval.
