# Supplementary material for: Novel pretreatment nomograms based on pan-immune-inflammation value for predicting clinical outcome in patients with head and neck squamous cell carcinoma
Source: Front Oncol. 2024 Jun 10;14:1399047. doi: 10.3389/fonc.2024.1399047 (PMC11194608; doi:10.3389/fonc.2024.1399047)
Supplement: Supplementary file 2 [file Table_2.docx]

**Supplementary Table 2**

Associations between PIV (stratified by cut-off 123.3) and HR for OS in various subgroups of the development cohort.

| **Characteristic** | **HR (95% CI)** | ***p*-value** | ***p* for interaction** |
| --- | --- | --- | --- |
| Age (year) |  |  | 0.378 |
| <60 | 4.138 (1.639-10.444) | 0.003 |  |
| ≥60 | 7.425 (3.843-14.346) | <0.001 |  |
| Smoking index |  |  | 0.129 |
| <650 | 7.646 (3.660-15.975) | <0.001 |  |
| ≥650 | 3.260 (1.515-7.016) | 0.003 |  |
| T stage |  |  | 0.426 |
| Tis/T1 | 8.525 (3.052-23.819) | <0.001 |  |
| T2 | 3.786 (1.391-10.303) | 0.009 |  |
| T3 | 3.280 (1.123-9.577) | 0.030 |  |
| T4 | 1.876 (0.478-7.367) | 0.367 |  |
| N stage |  |  | 0.031 |
| N0 | 10.449 (4.390-24.867) | <0.001 |  |
| N1 | 15.779 (1.809-137.636) | 0.013 |  |
| N2 | 2.099 (0.869-5.070) | 0.099 |  |
| TNM stage (AJCC, 8th) |  |  | 0.052 |
| 0/I | 19.142 (4.300-85.208) | <0.001 |  |
| II | 2.511 (0.705-9.233) | 0.154 |  |
| III | 3.489 (1.238-9.835) | 0.018 |  |
| IV | 2.291 (1.008-5.209) | 0.048 |  |
| PORT/POCRT |  |  | 0.024 |
| Undone | 8.735 (3.837-19.886) | <0.001 |  |
| Done | 2.823 (1.395-5.710) | 0.004 |  |
| FIB |  |  | 0.655 |
| Normal | 5.933 (3.184-11.054) | <0.001 |  |
| Abnormal | 4.214 (1.519-11.689) | 0.006 |  |
| ALB |  |  | 0.320 |
| Normal | 6.925 (2.985-16.064) | <0.001 |  |
| Abnormal | 4.396 (2.219-8.707) | <0.001 |  |
| TBIL |  |  | 0.615 |
| Normal | 5.175 (2.949-9.081) | <0.001 |  |
| Abnormal | 8.237 (1.686-40.250) | 0.009 |  |
|  |  |  |  |

Abbreviations: OS, overall survival.
