# Supplementary material for: Novel pretreatment nomograms based on pan-immune-inflammation value for predicting clinical outcome in patients with head and neck squamous cell carcinoma
Source: Front Oncol. 2024 Jun 10;14:1399047. doi: 10.3389/fonc.2024.1399047 (PMC11194608; doi:10.3389/fonc.2024.1399047)
Supplement: Supplementary file 3 [file Table_3.docx]

**Supplementary Table 3**

Univariate and multivariate analyses of OS according to clinicopathological factors in the development cohort.

| **Characteristic** | **Univariate analysis** | |  | **Multivariate analysis** | |
| --- | --- | --- | --- | --- | --- |
|  | **HR (95% CI)** | ***p*-value** |  | **HR (95% CI)** | ***p*-value** |
| Sex |  |  |  |  |  |
| Female | Ref |  |  |  |  |
| Male | 2.433 (0.598-9.901) | 0.214 |  |  |  |
| Age (year) |  |  |  |  |  |
| <60 | Ref |  |  |  |  |
| ≥60 | 2.171 (1.345-3.506) | 0.002 |  | 2.065 (1.252-3.406) | 0.004 |
| Smoking index |  |  |  |  |  |
| <650 | Ref |  |  |  |  |
| ≥650 | 1.715 (1.102-2.670) | 0.017 |  |  |  |
| Tumor type |  |  |  |  |  |
| Laryngeal cancer | Ref | 0.679 |  |  |  |
| Hypopharyngeal cancer | 0.829 (0.414-1.662) | 0.598 |  |  |  |
| Other types | 0.591 (0.145-2.412) | 0.464 |  |  |  |
| Tumor differentiation |  |  |  |  |  |
| Well differentiated | Ref | 0.215 |  |  |  |
| Moderately differentiated | 0.920 (0.569-1.487) | 0.734 |  |  |  |
| Poorly differentiated | 1.623 (0.859-3.063) | 0.136 |  |  |  |
| T stage |  |  |  |  |  |
| Tis/T1 | Ref | <0.001 |  |  |  |
| T2 | 4.014 (2.190-7.358) | <0.001 |  |  |  |
| T3 | 4.617 (2.570-8.295) | <0.001 |  |  |  |
| T4 | 7.275 (3.273-16.171) | <0.001 |  |  |  |
| N stage |  |  |  |  |  |
| N0 | Ref | <0.001 |  |  |  |
| N1 | 2.548 (1.311-4.954) | 0.006 |  |  |  |
| N2 | 2.773 (1.698-4.527) | <0.001 |  |  |  |
| TNM stage (AJCC, 8th) |  |  |  |  |  |
| 0/I | Ref | <0.001 |  | Ref | <0.001 |
| II | 4.124 (1.987-8.560) | <0.001 |  | 2.815 (1.312-6.041) | 0.008 |
| III | 5.078 (2.591-9.952) | <0.001 |  | 3.624 (1.827-7.188) | <0.001 |
| IV | 6.633 (3.563-12.350) | <0.001 |  | 3.925 (2.073-7.434) | <0.001 |
| PORT/POCRT |  |  |  |  |  |
| Undone | Ref |  |  |  |  |
| Done | 2.499 (1.629-3.834) | <0.001 |  |  |  |
| FIB |  |  |  |  |  |
| Normal | Ref |  |  |  |  |
| Abnormal | 2.257 (1.379-3.696) | 0.001 |  |  |  |
| ALB |  |  |  |  |  |
| Normal | Ref |  |  |  |  |
| Abnormal | 1.681 (1.076-2.626) | 0.022 |  |  |  |

**Supplementary Table 3** (*continued*)

| **Characteristic** | **Univariate analysis** | |  | **Multivariate analysis** | |
| --- | --- | --- | --- | --- | --- |
|  | **HR (95% CI)** | ***p*-value** |  | **HR (95% CI)** | ***p*-value** |
| TBIL |  |  |  |  |  |
| Normal | Ref |  |  |  |  |
| Abnormal | 0.765 (0.382-1.530) | 0.448 |  |  |  |
| NLR | 1.325 (1.213-1.448) | <0.001 |  | 1.196 (1.080-1.324) | 0.001 |
| PLR | 1.009 (1.005-1.013) | <0.001 |  |  |  |
| LMR | 0.651 (0.560-0.757) | <0.001 |  |  |  |
| PIV |  |  |  |  |  |
| Low (<123.3) | Ref |  |  |  |  |
| High (≥123.3) | 5.546 (3.263-9.426) | <0.001 |  | 3.600 (2.053-6.314) | <0.001 |
