# Supplementary material for: Novel pretreatment nomograms based on pan-immune-inflammation value for predicting clinical outcome in patients with head and neck squamous cell carcinoma
Source: Front Oncol. 2024 Jun 10;14:1399047. doi: 10.3389/fonc.2024.1399047 (PMC11194608; doi:10.3389/fonc.2024.1399047)
Supplement: Supplementary file 4 [file Table_4.docx]

**Supplementary Table 4**

The association between PIV and HR for DFS according to Model A and Model B in the development cohort.

| **Pan-Immune-Inflammation Value**  **(PIV)** | **Model A** | |  | **Model B** | |
| --- | --- | --- | --- | --- | --- |
|  | **HR (95% CI)** | ***p*-value** |  | **HR (95% CI)** | ***p*-value** |
| As continuous (per SD) | 1.003 (1.002-1.004) | <0.001 |  | 1.003 (1.002-1.004) | <0.001 |
| By PIV cut-off |  |  |  |  |  |
| Low (<123.3) | Ref |  |  | Ref |  |
| High (≥123.3) | 3.605 (2.109-6.162) | <0.001 |  | 2.569 (1.417-4.657) | 0.002 |
| Interquartile |  |  |  |  |  |
| Q1 (<83.6) | Ref | <0.001 |  | Ref | 0.024 |
| Q2 (83.6-135.1) | 1.602 (0.699-3.672) | 0.265 |  | 1.271 (0.541-2.983) | 0.582 |
| Q3 (135.1-204.1) | 3.826 (1.753-8.350) | 0.001 |  | 2.219 (0.945-5.208) | 0.067 |
| Q4 (≥204.1) | 4.162 (1.887-9.183) | <0.001 |  | 3.090 (1.295-7.374) | 0.011 |
| *p* for trend |  | <0.001 |  |  | <0.001 |

Note: Model A was adjusted for age, TNM stage, NLR and PIV. Model B was adjusted for TNM stage, LMR and PIV.
