# Supplementary material for: Novel pretreatment nomograms based on pan-immune-inflammation value for predicting clinical outcome in patients with head and neck squamous cell carcinoma
Source: Front Oncol. 2024 Jun 10;14:1399047. doi: 10.3389/fonc.2024.1399047 (PMC11194608; doi:10.3389/fonc.2024.1399047)
Supplement: Supplementary file 5 [file Table_5.docx]

**Supplementary Table 5**

The association between PIV and HR for OS according to Model A and Model B in the development cohort.

| **Pan-Immune-Inflammation Value**  **(PIV)** | **Model A** | |  | **Model B** | |
| --- | --- | --- | --- | --- | --- |
|  | **HR (95% CI)** | ***p*-value** |  | **HR (95% CI)** | ***p*-value** |
| As continuous (per SD) | 1.004 (1.002-1.005) | <0.001 |  | 1.003 (1.002-1.004) | <0.001 |
| By PIV cut-off |  |  |  |  |  |
| Low (<123.3) | Ref |  |  | Ref |  |
| High (≥123.3) | 3.600 (2.053-6.314) | <0.001 |  | 2.469 (1.333-4.573) | 0.004 |
| Interquartile |  |  |  |  |  |
| Q1 (<83.6) | Ref | 0.001 |  | Ref | 0.033 |
| Q2 (83.6-135.1) | 1.366 (0.582-3.204) | 0.473 |  | 1.048 (0.435-2.524) | 0.917 |
| Q3 (135.1-204.1) | 3.261 (1.478-7.191) | 0.003 |  | 1.732 (0.728-4.120) | 0.215 |
| Q4 (≥204.1) | 3.747 (1.692-8.301) | 0.001 |  | 2.658 (1.112-6.352) | 0.028 |
| *p* for trend |  | <0.001 |  |  | 0.007 |
