# Supplementary material for: Novel pretreatment nomograms based on pan-immune-inflammation value for predicting clinical outcome in patients with head and neck squamous cell carcinoma
Source: Front Oncol. 2024 Jun 10;14:1399047. doi: 10.3389/fonc.2024.1399047 (PMC11194608; doi:10.3389/fonc.2024.1399047)
Supplement: Supplementary file 6 [file Table_6.docx]

**Supplementary Table 6**

Baseline clinicopathological characteristics of the RT/CRT cohort.

| **Characteristic** | **Total Patients** | **PIV-Low** | **PIV-High** | ***p*-value** |
| --- | --- | --- | --- | --- |
| N (%) | 50 | 14 (28.0) | 36 (72.0) |  |
| Sex, N (%) |  |  |  | 0.916 |
| Male | 45 (90.0) | 12 (85.7) | 33 (91.7) |  |
| Female | 5 (10.0) | 2 (14.3) | 3 (8.3) |  |
| Age (year), N (%) |  |  |  | 0.363 |
| <60 | 27 (54.0) | 9 (64.3) | 18 (50.0) |  |
| ≥60 | 23 (46.0) | 5 (35.7) | 18 (50.0) |  |
| Smoking index, N (%) |  |  |  | 0.066 |
| <650 | 29 (58.0) | 11 (78.6) | 18 (50.0) |  |
| ≥650 | 21 (42.0) | 3 (21.4) | 18 (50.0) |  |
| Tumor type, N (%) |  |  |  | 0.619 |
| Laryngeal cancer | 30 (60.0) | 8 (57.1) | 22 (61.1) |  |
| Hypopharyngeal cancer | 16 (32.0) | 4 (28.6) | 12 (33.3) |  |
| Other types | 4 (8.0) | 2 (14.3) | 2 (5.6) |  |
| Tumor differentiation, N (%) |  |  |  | 0.225 |
| Well differentiated | 11 (22.0) | 5 (35.7) | 6 (16.7) |  |
| Moderately differentiated | 27 (54.0) | 5 (35.7) | 22 (61.1) |  |
| Poorly differentiated | 12 (24.0) | 4 (28.6) | 8 (22.2) |  |
| T stage, N (%) |  |  |  | <0.001 |
| Tis/T1 | 12 (24.0) | 9 (64.3) | 3 (8.3) |  |
| T2 | 15 (30.0) | 4 (28.6) | 11 (30.6) |  |
| T3 | 17 (34.0) | 1 (7.1) | 16 (44.4) |  |
| T4 | 6 (12.0) | 0 (0) | 6 (16.7) |  |
| N stage, N (%) |  |  |  | 0.003 |
| N0 | 30 (60.0) | 13 (92.9) | 17 (47.2) |  |
| N1 | 10 (20.0) | 1 (7.1) | 9 (25.0) |  |
| N2 | 10 (20.0) | 0 (0) | 10 (27.8) |  |
| M stage, N (%) |  |  |  | 0.035 |
| M0 | 38 (76.0) | 14 (100.0) | 24 (66.7) |  |
| M1 | 12 (24.0) | 0 (0) | 12 (33.3) |  |
| TNM stage (AJCC, 8th), N (%) |  |  |  | <0.001 |
| 0/I | 11 (22.0) | 9 (64.3) | 2 (5.6) |  |
| II | 10 (20.0) | 3 (21.4) | 7 (19.4) |  |
| III | 13 (26.0) | 2 (14.3) | 11 (30.6) |  |
| IV | 16 (32.0) | 0 (0) | 16 (44.4) |  |
| FIB, N (%) |  |  |  | <0.001 |
| Normal | 31 (62.0) | 14 (100.0) | 17 (47.2) |  |
| Abnormal | 19 (38.0) | 0 (0) | 19 (52.8) |  |
| ALB, N (%) |  |  |  | 0.066 |
| Normal | 29 (58.0) | 11 (78.6) | 18 (50.0) |  |

**Supplementary Table 6** (*continued*)

| **Characteristic** | **Total Patients** | **PIV-Low** | **PIV-High** | ***p*-value** |
| --- | --- | --- | --- | --- |
| Abnormal | 21 (42.0) | 3 (21.4) | 18 (50.0) |  |
| TBIL, N (%) |  |  |  | 0.246 |
| Normal | 48 (96.0) | 14 (100.0) | 34 (94.4) |  |
| Abnormal | 2 (4.0) | 0 (0) | 2 (5.6) |  |
| LYM (10^9^/L), mean ± SD | 1.48 ± 0.74 | 1.83 ± 0.78 | 1.34 ± 0.69 | 0.035 ^#^ |
| MON (10^9^/L), median (IQR) | 0.36 (0.28-0.51) | 0.33 (0.19-0.42) | 0.44 (0.29-0.56) | 0.082 ⃰ |
| NEU (10^9^/L), median (IQR) | 5.06 (3.57-7.17) | 2.90 (1.98-3.78) | 6.09 (3.42-8.13) | <0.001 ⃰ |
| PLT (10^9^/L), mean ± SD | 233.36 ± 77.29 | 196.07 ± 59.86 | 247.86 ± 79.12 | <0.001 ^#^ |
| NLR, median (IQR) | 3.36 (1.97-9.51) | 1.40 (1.15-2.12) | 5.31 (2.94-10.82) | <0.001 ⃰ |
| PLR, median (IQR) | 150.03 (117.89-260.08) | 108.27 (80.87-153.11) | 179.74 (135.79-316.95) | 0.001 ⃰ |
| LMR, median (IQR) | 4.01 (2.33-5.63) | 5.72 (4.54-9.02) | 3.17 (2.55-4.35) | <0.001 ⃰ |

Note: ⃰ Wilcoxon rank sum test, ^#^ one-way analysis of variance, others are Chi-square test. Data are represented as mean (SD), median (interquartile range) or number (%).
