# Supplementary material for: Novel pretreatment nomograms based on pan-immune-inflammation value for predicting clinical outcome in patients with head and neck squamous cell carcinoma
Source: Front Oncol. 2024 Jun 10;14:1399047. doi: 10.3389/fonc.2024.1399047 (PMC11194608; doi:10.3389/fonc.2024.1399047)
Supplement: Supplementary file 7 [file Table_7.docx]

**Supplementary Table 7**

Univariate and multivariate analyses of PFS according to clinicopathological factors in the RT/CRT cohort.

| **Characteristic** | **Univariate analysis** | |  | **Multivariate analysis** | |
| --- | --- | --- | --- | --- | --- |
|  | **HR (95% CI)** | ***p*-value** |  | **HR (95% CI)** | ***p*-value** |
| Sex |  |  |  |  |  |
| Female | Ref |  |  |  |  |
| Male | 1.407 (0.498-3.977) | 0.519 |  |  |  |
| Age (year) |  |  |  |  |  |
| <60 | Ref |  |  |  |  |
| ≥60 | 1.487 (0.779-2.839) | 0.229 |  |  |  |
| Smoking index |  |  |  |  |  |
| <650 | Ref |  |  |  |  |
| ≥650 | 2.621 (1.342-5.119) | 0.005 |  |  |  |
| Tumor type |  |  |  |  |  |
| Laryngeal cancer | Ref | 0.746 |  |  |  |
| Hypopharyngeal cancer | 1.020 (0.513-2.030) | 0.954 |  |  |  |
| Other types | 1.605 (0.473-5.449) | 0.448 |  |  |  |
| Tumor differentiation |  |  |  |  |  |
| Well differentiated | Ref | 0.202 |  |  |  |
| Moderately differentiated | 2.233 (0.899-5.546) | 0.084 |  |  |  |
| Poorly differentiated | 1.581 (0.573-4.359) | 0.376 |  |  |  |
| T stage |  |  |  |  |  |
| Tis/T1 | Ref | 0.005 |  |  |  |
| T2 | 5.823 (1.829-18.539) | 0.003 |  |  |  |
| T3 | 6.790 (2.208-20.882) | 0.001 |  |  |  |
| T4 | 9.745 (2.545-37.309) | 0.001 |  |  |  |
| N stage |  |  |  |  |  |
| N0 | Ref | 0.001 |  |  |  |
| N1 | 3.285 (1.381-7.815) | 0.007 |  |  |  |
| N2 | 4.915 (2.001-12.075) | 0.001 |  |  |  |
| M stage |  |  |  |  |  |
| M0 | Ref |  |  |  |  |
| M1 | 3.791 (1.702-8.446) | 0.001 |  |  |  |
| TNM stage (AJCC, 8th) |  |  |  |  |  |
| 0/I | Ref | <0.001 |  | Ref | 0.017 |
| II | 6.469 (1.665-25.129) | 0.007 |  | 6.219 (1.363-28.380) | 0.018 |
| III | 8.584 (2.342-31.462) | 0.001 |  | 9.738 (1.905-49.768) | 0.006 |
| IV | 19.680 (4.994-77.559) | <0.001 |  | 13.945 (2.750-70.728) | 0.001 |
| FIB |  |  |  |  |  |
| Normal | Ref |  |  |  |  |
| Abnormal | 3.433 (1.765-6.675) | <0.001 |  |  |  |
| ALB |  |  |  |  |  |
| Normal | Ref |  |  |  |  |
| Abnormal | 2.129 (1.110-4.081) | 0.023 |  |  |  |

**Supplementary Table 7** (*continued*)

| **Characteristic** | **Univariate analysis** | |  | **Multivariate analysis** | |
| --- | --- | --- | --- | --- | --- |
|  | **HR (95% CI)** | ***p*-value** |  | **HR (95% CI)** | ***p*-value** |
| TBIL |  |  |  |  |  |
| Normal | Ref |  |  |  |  |
| Abnormal | 0.994 (0.238-4.145) | 0.993 |  |  |  |
| NLR | 1.083 (1.044-1.124) | <0.001 |  |  |  |
| PLR | 1.004 (1.003-1.006) | <0.001 |  | 1.007 (1.003-1.012） | 0.002 |
| LMR | 0.796 (0.684-0.926) | 0.003 |  |  |  |
| PIV |  |  |  |  |  |
| Low (<123.3) | Ref |  |  |  |  |
| High (≥123.3) | 12.486 (3.759-41.474) | <0.001 |  | 4.890 (1.223-19.545) | 0.025 |
