# Supplementary material for: Novel pretreatment nomograms based on pan-immune-inflammation value for predicting clinical outcome in patients with head and neck squamous cell carcinoma
Source: Front Oncol. 2024 Jun 10;14:1399047. doi: 10.3389/fonc.2024.1399047 (PMC11194608; doi:10.3389/fonc.2024.1399047)
Supplement: Supplementary file 8 [file Table_8.docx]

**Supplementary Table 8**

Univariate and multivariate analyses of OS according to clinicopathological factors in the RT/ CRT cohort.

| **Characteristic** | **Univariate analysis** | |  | **Multivariate analysis** | |
| --- | --- | --- | --- | --- | --- |
|  | **HR (95% CI)** | ***p*-value** |  | **HR (95% CI)** | ***p*-value** |
| Sex |  |  |  |  |  |
| Female | Ref |  |  |  |  |
| Male | 1.046 (0.363-3.016) | 0.934 |  |  |  |
| Age (year) |  |  |  |  |  |
| <60 | Ref |  |  |  |  |
| ≥60 | 1.514 (0.768-2.983) | 0.231 |  |  |  |
| Smoking index |  |  |  |  |  |
| <650 | Ref |  |  |  |  |
| ≥650 | 2.981 (1.514-5.869) | 0.002 |  | 2.387 (1.130-5.039) | 0.023 |
| Tumor type |  |  |  |  |  |
| Laryngeal cancer | Ref | 0.366 |  |  |  |
| Hypopharyngeal cancer | 1.271 (0.611-2.642) | 0.521 |  |  |  |
| Other types | 2.394 (0.695-8.254) | 0.167 |  |  |  |
| Tumor differentiation |  |  |  |  |  |
| Well differentiated | Ref | 0.482 |  |  |  |
| Moderately differentiated | 1.703 (0.679-4.272) | 0.257 |  |  |  |
| Poorly differentiated | 1.756 (0.631-4.884) | 0.281 |  |  |  |
| T stage |  |  |  |  |  |
| Tis/T1 | Ref | 0.003 |  |  |  |
| T2 | 7.152 (2.156-23.725) | 0.001 |  |  |  |
| T3 | 9.227 (2.697-31.563) | <0.001 |  |  |  |
| T4 | 9.637 (2.529-36.720) | 0.001 |  |  |  |
| N stage |  |  |  |  |  |
| N0 | Ref | 0.005 |  |  |  |
| N1 | 3.789 (1.513-9.488) | 0.004 |  |  |  |
| N2 | 2.864 (1.256-6.531) | 0.012 |  |  |  |
| M stage |  |  |  |  |  |
| M0 | Ref |  |  |  |  |
| M1 | 3.645 (1.626-8.174) | 0.002 |  |  |  |
| TNM stage (AJCC, 8th) |  |  |  |  |  |
| 0/I | Ref | 0.001 |  | Ref | 0.007 |
| II | 8.048 (1.996-32.446) | 0.003 |  | 5.203 (1.091-24.825) | 0.039 |
| III | 13.443 (3.189-56.676) | <0.001 |  | 19.505 (3.420-111.256) | 0.001 |
| IV | 17.042 (4.345-66.839) | <0.001 |  | 14.773 (3.017-72.332) | 0.001 |
| FIB |  |  |  |  |  |
| Normal | Ref |  |  |  |  |
| Abnormal | 3.660 (1.811-7.398) | <0.001 |  |  |  |
| ALB |  |  |  |  |  |
| Normal | Ref |  |  |  |  |
| Abnormal | 2.668 (1.338-5.320) | 0.005 |  |  |  |

**Supplementary Table 8** (*continued*)

| **Characteristic** | **Univariate analysis** | |  | **Multivariate analysis** | |
| --- | --- | --- | --- | --- | --- |
|  | **HR (95% CI)** | ***p*-value** |  | **HR (95% CI)** | ***p*-value** |
| TBIL |  |  |  |  |  |
| Normal | Ref |  |  |  |  |
| Abnormal | 1.615 (0.382-6.828) | 0.515 |  |  |  |
| NLR | 1.083 (1.044-1.123) | <0.001 |  |  |  |
| PLR | 1.004 (1.002-1.006) | <0.001 |  | 1.008 (1.003-1.013） | 0.003 |
| LMR | 0.811 (0.694-0.948) | 0.008 |  |  |  |
| PIV |  |  |  |  |  |
| Low (<123.3) | Ref |  |  |  |  |
| High (≥123.3) | 13.222 (3.851-45.395) | <0.001 |  | 5.581 (1.239-25.140) | 0.025 |
