# Supplementary figures and images for: Novel pretreatment nomograms based on pan-immune-inflammation value for predicting clinical outcome in patients with head and neck squamous cell carcinoma
Source: Front Oncol. 2024 Jun 10;14:1399047. doi: 10.3389/fonc.2024.1399047 (PMC11194608; doi:10.3389/fonc.2024.1399047)

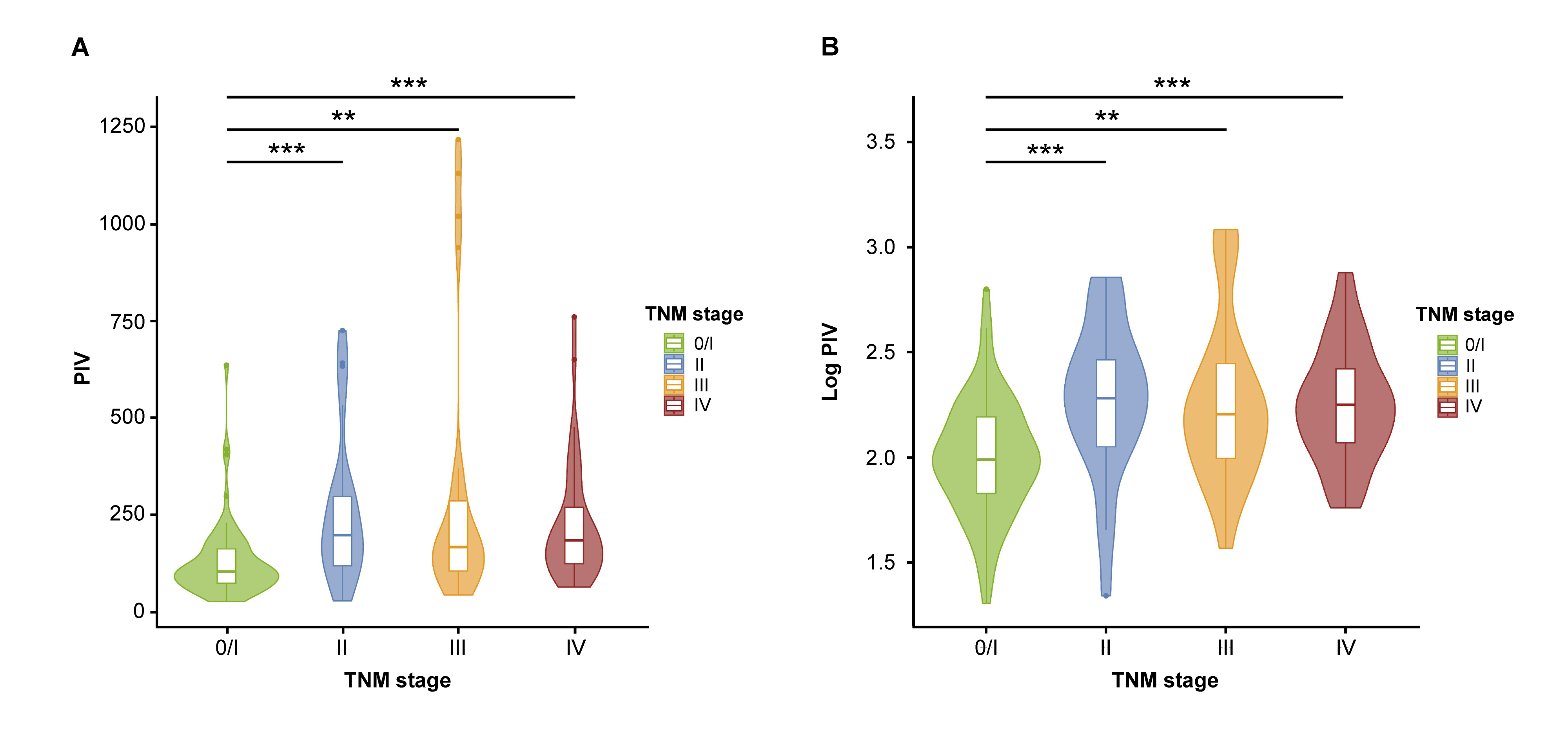

Supplement: Supplementary Figure 1 — Relationships between PIV and TNM stages in the development cohort. (A) The relationship between PIV and different TNM stages. (B) The relationship between Log PIV (natural-log transformed) and different TNM stages. **p<0.01; ***p<0.001. PIV, Pan-Immune-Inflammation value. [file Image_1.tif]

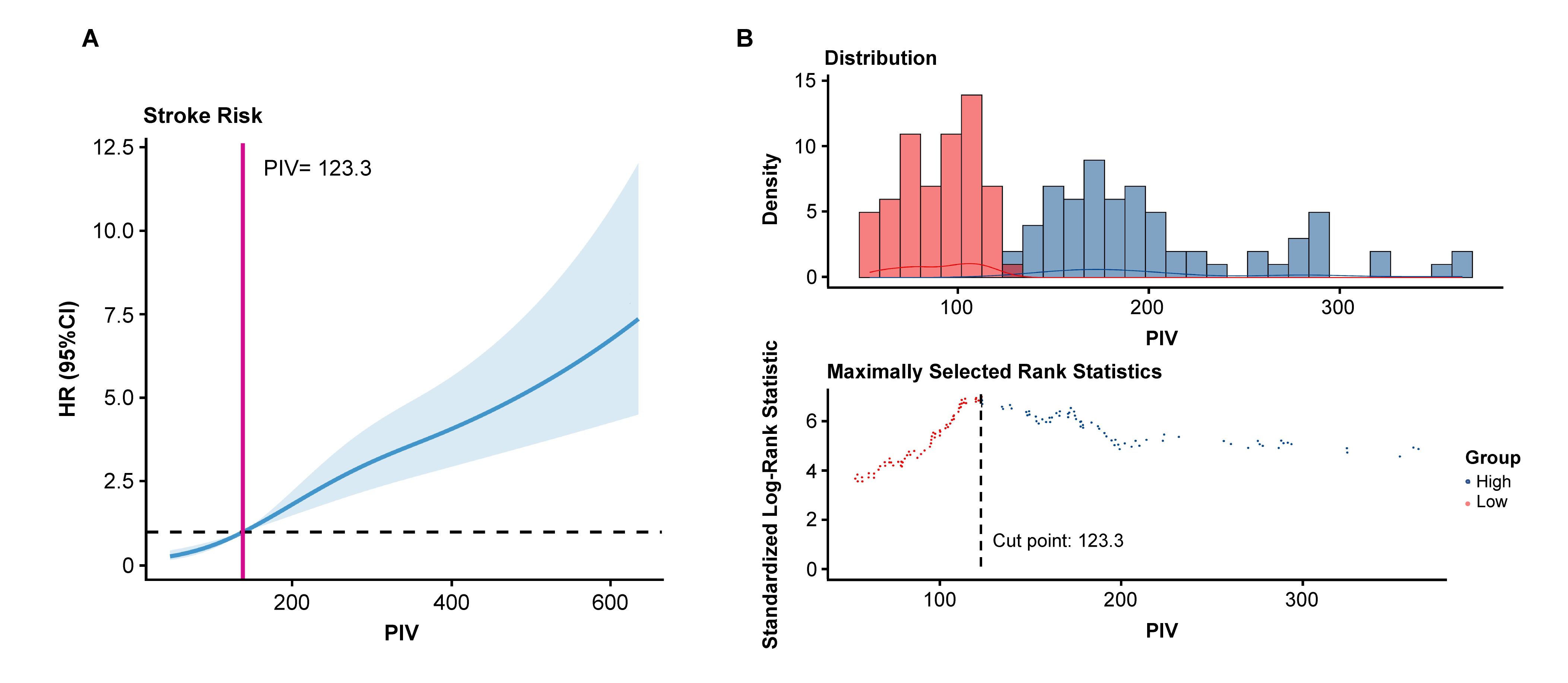

Supplement: Supplementary Figure 2 — Association of PIV with DFS in the development cohort. (A) The association between PIV (continuous) and hazard ratio of DFS based on restricted cubic spline plot. (B) The cut-off for PIV associated with DFS based on maximally selected rank statistics. DFS, disease-free survival; HR, hazard ratio. [file Image_2.tif]

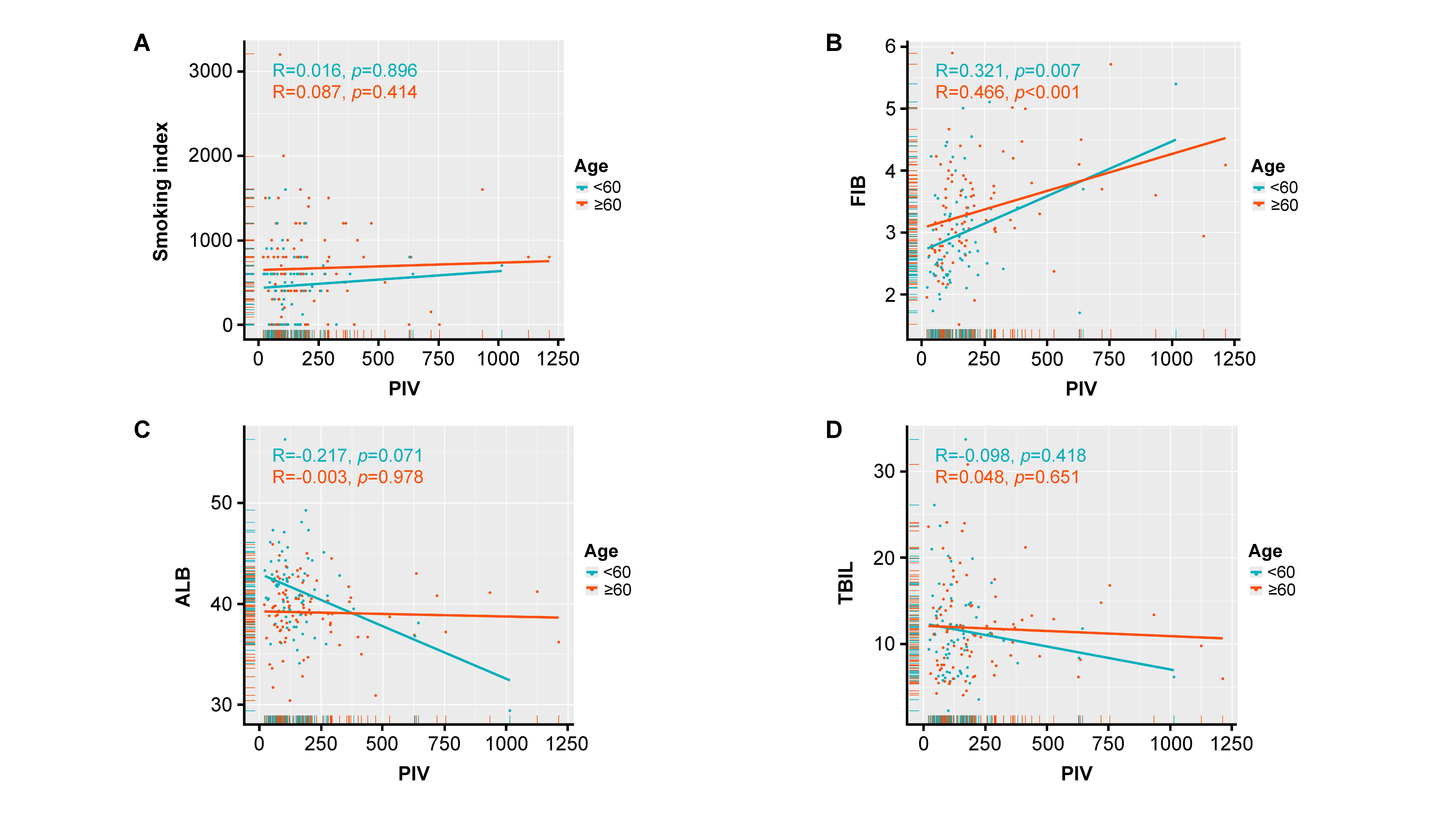

Supplement: Supplementary Figure 3 — Relationships between PIV and clinical parameters in the development cohort. The clinical parameters include smoking index (A), FIB (B), ALB (C) and TBIL (D), respectively. FIB, fibrinogen; ALB, albumin; TBIL, total bilirubin. [file Image_3.tif]

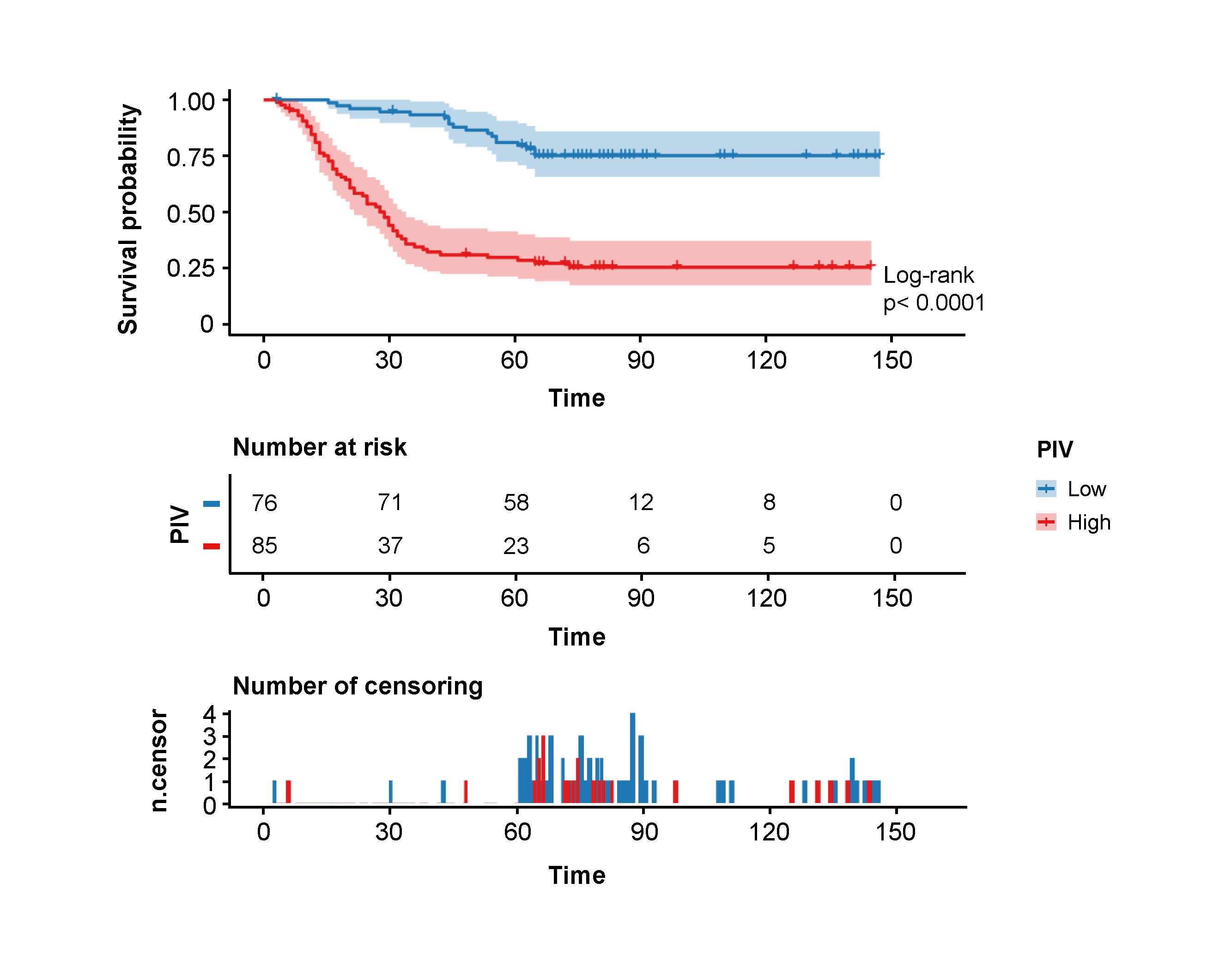

Supplement: Supplementary Figure 4 — Kaplan-Meier survival analysis of OS in the different PIV (Low and High) groups of the development cohort. OS, overall survival. [file Image_4.tif]

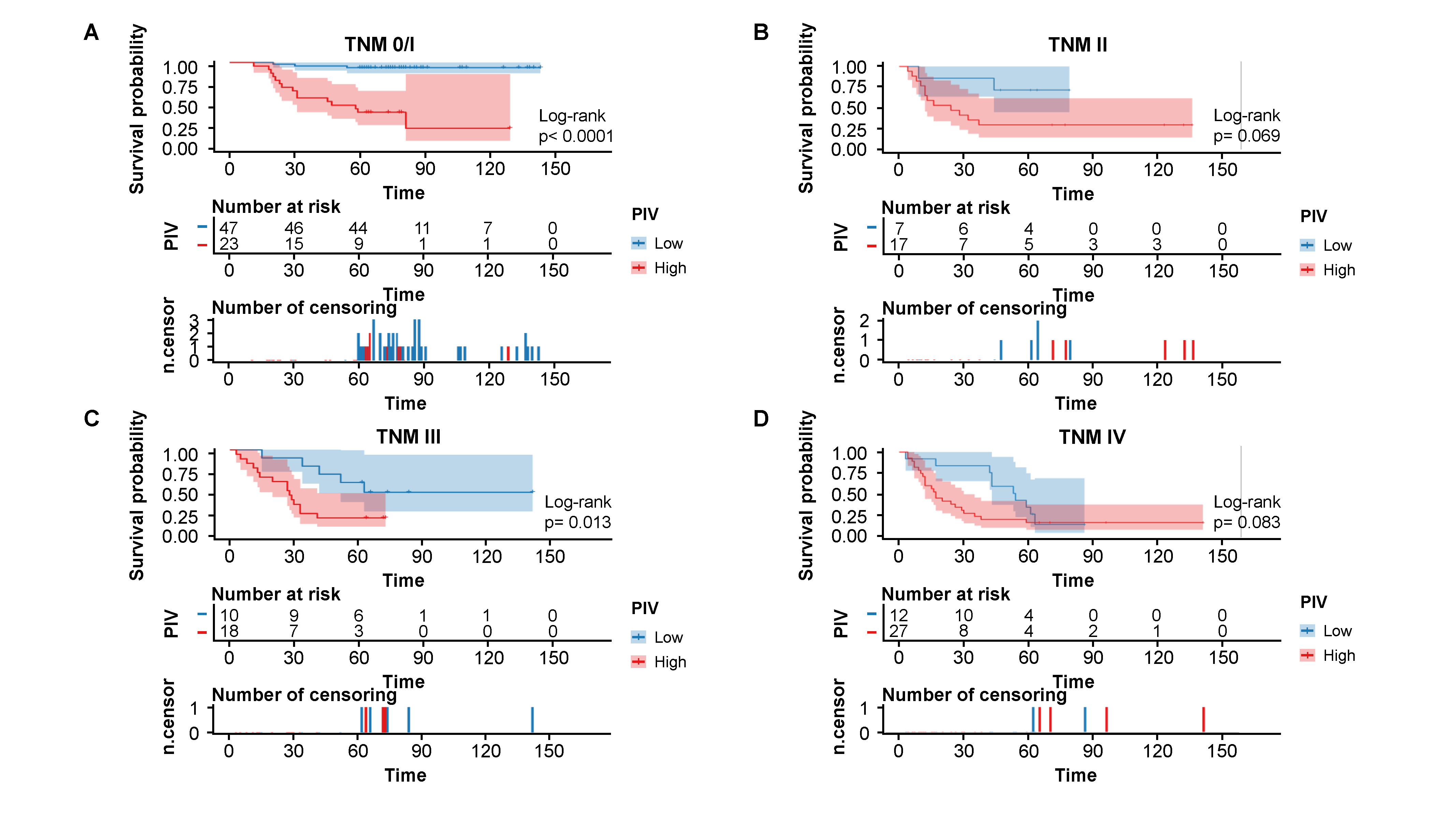

Supplement: Supplementary Figure 5 — Subgroup Kaplan-Meier survival analyses of DFS of the development cohort in the different PIV (Low and High) groups. All patients were stratified into four subgroups including TNM stage 0/I (A), stage II (B), stage III (C) and stage IV (D). [file Image_5.tif]

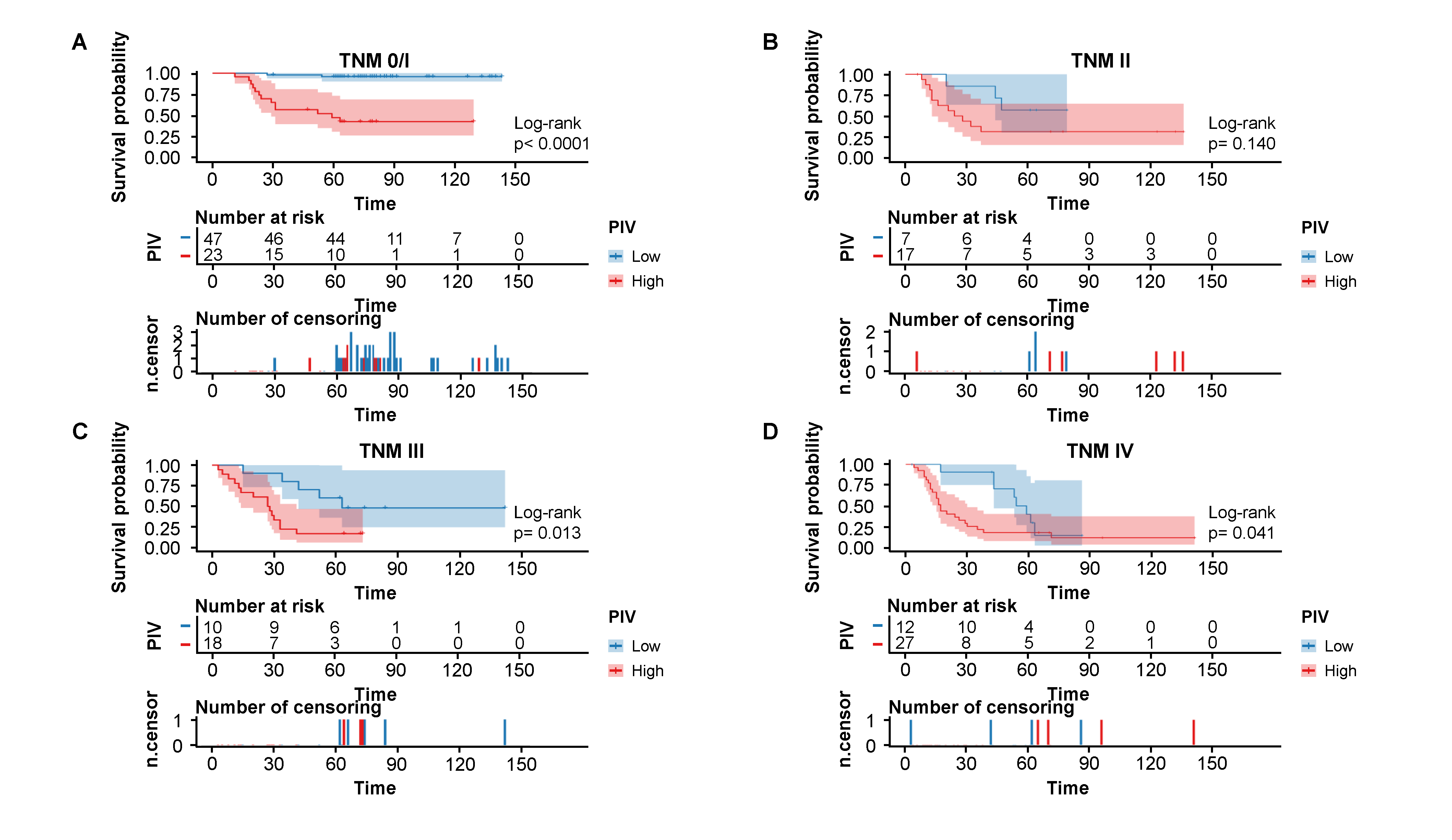

Supplement: Supplementary Figure 6 — Subgroup Kaplan-Meier survival analyses of OS of the development cohort in the different PIV (Low and High) groups. All patients were stratified into four subgroups including TNM stage 0/I (A), stage II (B), stage III (C) and stage IV (D). [file Image_6.tif]

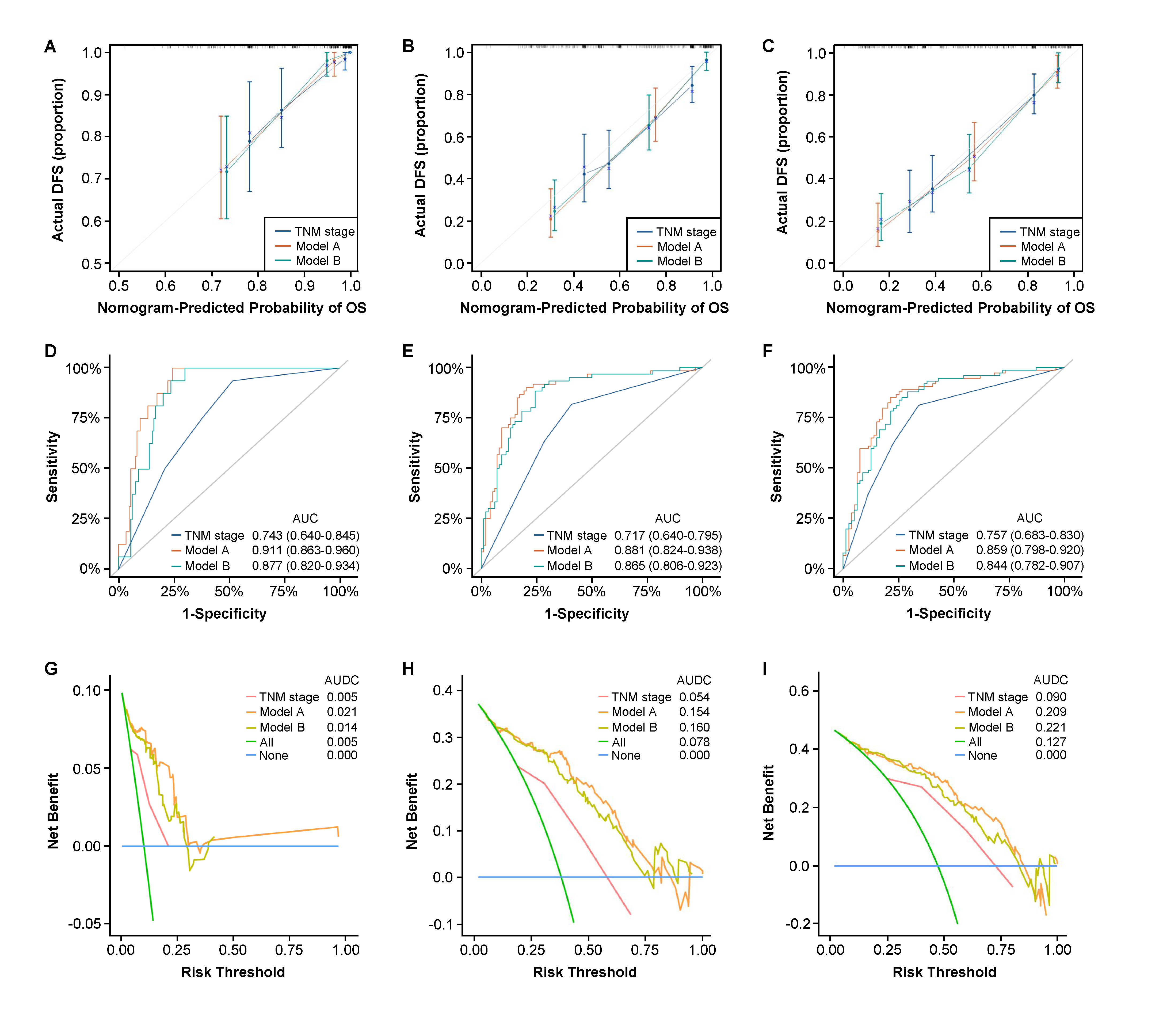

Supplement: Supplementary Figure 7 — Nomograms internal validation according to bootstrapping method for 1, 3, 5-year OS in the development cohort. Calibration curves for 1-year (A), 3-year (B) and 5-year (C) OS prediction based on TNM stage, Model A and Model B. ROC analyses of 1-year (D), 3-year (E) and 5-year (F) OS prediction based on TNM stage, Model A and Model B. DCA curves for 1-year (G), 3-year (H) and 5-year (I) OS prediction based on TNM stage, Model A and Model B. [file Image_7.tif]

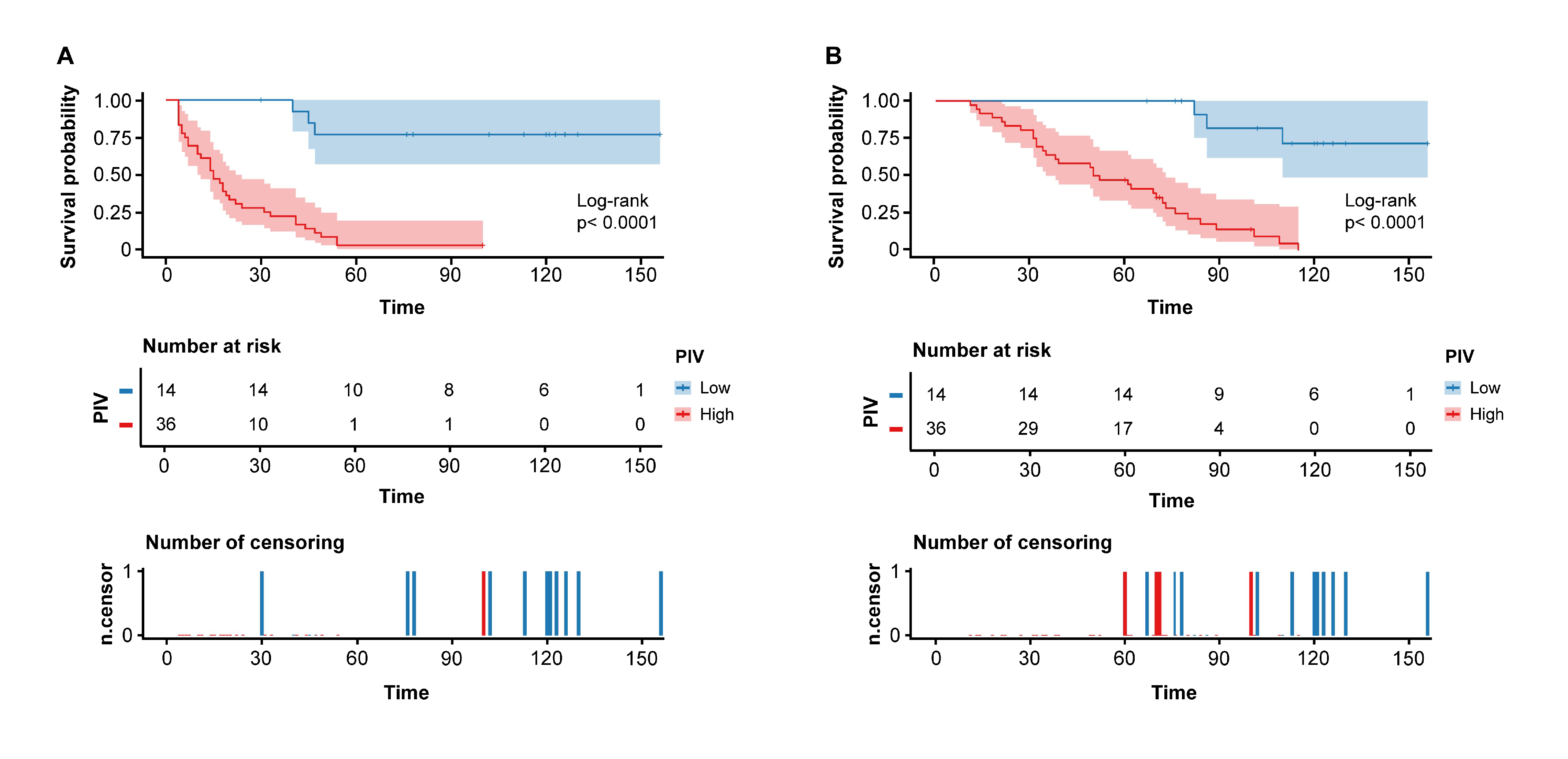

Supplement: Supplementary Figure 8 — Kaplan-Meier survival analyses of DFS (A) and OS (B) in the different PIV (Low and High) groups of the RT/CRT cohort. [file Image_8.tif]

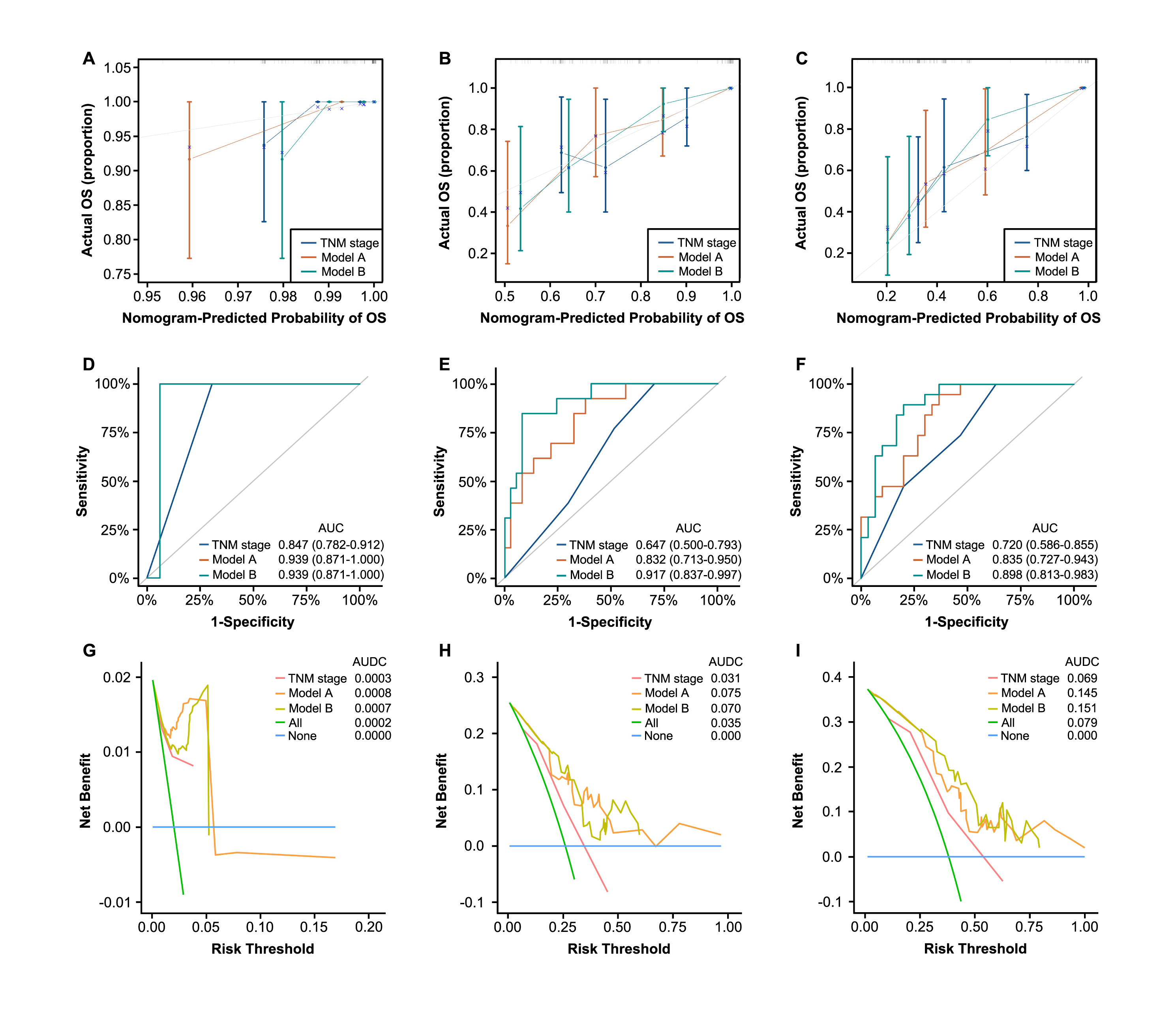

Supplement: Supplementary Figure 9 — Generality testing of nomograms for 1, 3, 5-year OS in the RT/CRT cohort. Calibration curves for 1-year (A), 3-year (B) and 5-year (C) OS prediction based on TNM stage, Model A and Model B. ROC analyses of 1-year (D), 3-year (E) and 5-year (F) OS prediction based on TNM stage, Model A and Model B. DCA curves for 1-year (G), 3-year (H) and 5-year (I) OS prediction based on TNM stage, Model A and Model B. [file Image_9.tif]
